# Recent Advancements in Polymer and Graphene-Based Composites for High-Performance Supercapacitor Applications: Progress, Challenges, and Future Perspectives

**DOI:** 10.3390/polym18212624

**Published:** 2026-10-23

**Authors:** Rajangam Vinodh, Raji Atchudan

**Affiliations:** 1 Division of Physical Sciences, Karunya Institute of Technology and Sciences (KITS), Karunya Nagar, Coimbatore 641114, Tamil Nadu, India; 2 School of Chemical Engineering, Yeungnam University, Gyeongsan 38541, Republic of Korea

**Keywords:** composite electrode, energy density, conducting polymers, supercapacitors, graphene, specific capacitance

## Abstract

The increasing worldwide need for sustainable and high-performance energy storage systems has accelerated significant interest in the development of advanced electrode materials for supercapacitors (SCs). SCs have attracted considerable attention as advanced energy storage devices because they offer high power output, rapid energy delivery and recharge, excellent rate capability, and outstanding stability over prolonged charge–discharge cycles, making them suitable for diverse energy storage and power-management applications. Nonetheless, the relatively low specific energy of conventional electrode materials remains a major obstacle to their widespread commercialization. In this context, polymers and graphene-derived electrode materials have gained substantial consideration owing to their complementary physicochemical characteristics. Conducting polymers (CPs), which include polyaniline (PANI), poly(3,4-ethylenedioxythiophene) (PEDOT), polypyrrole (PPy), and polythiophene (PTh) derivatives, and hypercross-linked polymers (HCPs) offer high pseudocapacitance through reversible redox reactions, whereas graphene and its derivatives provide extraordinary electrical conductivity, higher specific surface area (SSA), outstanding mechanical strength, and excellent chemical durability. The synergistic integration of these materials into polymer/graphene composites has begun as an efficient approach to overcome the intrinsic limitations of individual components and significantly improve electrochemical behavior. This review summarizes recent advances in polymer and graphene-derived composites for high-performance supercapacitor applications, focusing on preparation strategies, structural features, charge-storage mechanisms, and electrochemical properties of conducting polymer/graphene hybrids. The effects of morphology, porosity, surface area, electrical conductivity, mechanical flexibility, and ion transport on device performance are critically discussed. Recent progress in flexible and wearable supercapacitors, along with challenges related to cycling stability, graphene restacking, large-scale production, and device integration, is also highlighted. Furthermore, future perspectives, including multifunctional electrode design, sustainable materials, and artificial intelligence-assisted material development, are presented to provide insights into the design of next-generation supercapacitor electrodes.

## 1. Introduction

The rapid increase in global energy utilization, together with the gradual exhaustion of fossil-fuel resources and growing concerns about environmental degradation, has accelerated efforts toward the development and adoption of cleaner, renewable, and sustainable energy technologies [1,2]. Sustainable energy sources, specifically wind- and solar-based energy, are increasingly recognized as environmentally friendly alternatives to fossil fuels. However, the inherent intermittency and fluctuating power output of these sustainable resources necessitate the expansion of advanced energy storage systems capable of ensuring stable and uninterrupted energy delivery. Consequently, high-performance electrochemical energy storage devices have become indispensable components of modern energy infrastructures [3,4,5]. Among the available energy storage tools, SCs have emerged as promising energy storage devices because they offer outstanding specific power, rapid rates of charging and discharging, excellent cycling durability, and extended service life. Owing to these advantageous characteristics, supercapacitors are considered highly suitable for applications requiring rapid energy delivery, including compact electric vehicles, electronics, backup power systems, and hybrid energy storage devices [6,7,8,9,10].

SCs, commonly referred to as electrochemical capacitors, constitute a unique category of energy storage systems that combine characteristics of conventional dielectric capacitors and rechargeable batteries. These devices retain the high power-delivery capability and rapid charging–discharging behavior associated with capacitors, while offering considerably higher energy storage capacity than traditional capacitors. Depending on the primary charge-storage process, SCs are generally classified as electrical double-layer capacitors (EDLCs), pseudocapacitors (PCs), and hybrid supercapacitors (HSCs). Their overall electrochemical performance is primarily dictated by the characteristics of the electrode materials, including electrical conductivity, accessible surface area, pore architecture, surface chemistry, and redox activity [11,12,13].

Conducting polymers, including PANI, PPy, PEDOT, and PTh byproducts, have gained enormous interest as pseudocapacitive electrode materials due to their extraordinary theoretical capacitance, reversible Faradaic reactions, controllable electrical conductivity, and relatively simple synthesis procedures [14,15]. Despite these advantages, their practical utilization is often restricted by inadequate mechanical stability and gradual electrochemical degradation triggered by repeated expansion and contraction during continuous charge and discharge cycling, ultimately leading to capacitance fading and shortened device lifetime.

The distinctive physicochemical properties of graphene-derived materials, such as graphene oxide (GO) and reduced graphene oxide (rGO), have led to their extensive investigation as supercapacitor electrode materials. In particular, their efficient electron transport, high theoretical SSA, mechanical robustness, and remarkable resistance to chemical degradation contribute to their suitability for high-performance energy storage systems. These combined features make graphene-derived materials especially suitable for charge storage through electrical double-layer mechanisms [16,17,18,19,20]. Nevertheless, the tendency of individual graphene layers to interact through π–π stacking and van der Waals forces can result in severe sheet aggregation and restacking. Such structural rearrangement decreases the effective surface area available for electrochemical reactions, restricts electrolyte access to active regions, and creates barriers to efficient ion diffusion throughout the electrode.

To address the shortcomings associated with individual materials, extensive research has focused on combining conducting polymers with graphene to produce multifunctional composite electrodes. The complementary properties of conducting polymers and graphene may contribute to improvements in electrical conductivity, electrochemical accessibility, structural integrity, and charge-transfer behavior, depending on the composite architecture and interfacial characteristics. Reported improvements in specific capacitance, rate capability, energy density, and cycling stability vary among composites and should be interpreted in the context of their composition, electrode configuration, and experimental conditions.

**Figure 1:**
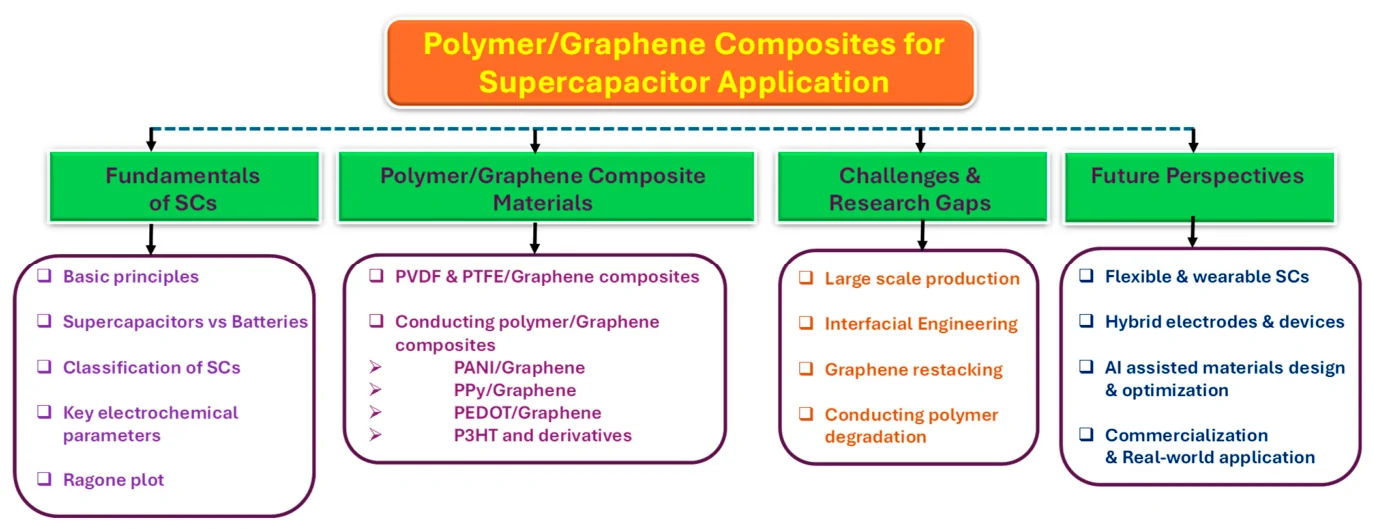
Schematic overview of the organization and major topics covered in this review on polymer/graphene-based composites for supercapacitor applications.

Emerging advances in the design of nanostructured materials, interface modification, and advanced synthesis methodologies have enabled the fabrication of polymer/graphene composites with precisely controlled architectures and optimized physicochemical properties. Although substantial progress has been achieved, several challenges, including scalable manufacturing, long-term operational stability, cost-effective production, and integration into practical energy storage devices, continue to impede their widespread commercialization. In this review, selected recent advances in polymer/graphene composite electrodes for supercapacitor applications are discussed, with particular attention given to synthesis techniques, structural design, electrochemical mechanisms, and performance optimization. In addition, the remaining scientific challenges, existing research gaps, and future prospects for the growth of next-generation polymer/graphene-based SCs are critically evaluated. Figure 1 illustrates the overall framework of this review on polymer/graphene composites for supercapacitor applications.

### 1.1. Scope and Distinguishing Features of This Review

Although numerous reviews have examined graphene-based, conducting polymer-based, and composite electrode materials for SC applications, the present review focuses on the interrelationship between polymer/graphene composite design, electrochemical charge storage behavior, and practical performance evaluation. Particular attention is given to conducting polymer/graphene-derived composites, including PANI, PPy, PEDOT, and related polymer architectures, together with graphene oxide and rGO. The review also considers polymeric binder–graphene systems, as well as ternary and quaternary composites incorporating transition metal oxides, metal sulfides, and MXene-derived components. The distinguishing feature of this review is its integrated discussion of material structure, composite interfaces, charge storage mechanisms, and electrochemical performance. The effects of graphene dispersion, sheet restacking, polymer–graphene interactions, electrical conductivity, surface accessibility, and ion transport on the behavior of composite electrodes are examined in relation to their reported electrochemical characteristics. In addition to summarizing synthesis approaches and materials properties, the review considers how the composition and architecture of composite electrodes contribute to specific capacitance, rate capability, energy density, and cycling stability.

Particular emphasis is placed on the importance of consistent electrochemical performance evaluation. Reported results are discussed with consideration of the measurement configuration, including three-electrode and two-electrode systems, the distinction between gravimetric, areal, and volumetric metrics, and the contribution of active material loading and device-level normalization. These considerations are important because performance values obtained under different experimental conditions may not be directly comparable. The present review therefore aims to provide a critical perspective on both the opportunities and limitations of polymer/graphene composites for practical supercapacitor applications.

Finally, the review addresses important challenges associated with the development of polymer/graphene-derived electrodes, including conducting polymer degradation, graphene restacking, interfacial compatibility, scalable synthesis, environmental considerations, and device integration. By connecting material design with electrochemical evaluation and practical implementation, this review provides a focused perspective on the progress and remaining research requirements for high-performance polymer/graphene composite supercapacitors.

### 1.2. Review Methodology and Literature Selection

A structured narrative literature search was conducted using Google, Google Scholar, and Web of Science, employing relevant keywords and combinations of terms related to graphene-based materials, conducting polymers, and supercapacitor applications. The search terms included “graphene”, “graphene oxide”, “reduced graphene oxide”, “conducting polymer”, “polyaniline”, “polypyrrole”, “polythiophene”, “supercapacitor”, “electrochemical capacitor”, and “energy storage”. Additional keyword combinations were used to identify the literature on graphene-based composites incorporating PVDF, PTFE, hypercross-linked polymers, MXene, metal oxides, metal sulfides, as well as flexible, wearable, and structural supercapacitors. The final literature search was performed on 23 June 2026, focusing primarily on publications from 2020 to 2026. English-language, peer-reviewed research articles and relevant review articles were considered. The relevance of the identified publications was assessed by examining their titles, abstracts, and, where appropriate, full texts, with particular attention to their relevance to the scope of this review and the availability of sufficient electrochemical information. Conference abstracts, patents, and duplicate records were not considered for the discussion.

This review follows a structured narrative approach rather than a formal systematic review protocol. Individual search logs and quantitative screening records were not maintained; therefore, retrospective database-specific screening counts and a quantitative screening flow cannot be reported reliably. Consequently, the review should not be interpreted as an exhaustive account of all publications on polymer/graphene composites for supercapacitor applications.

## 2. Fundamentals of Supercapacitors

### 2.1. Basic Principles of Supercapacitors

SCs constitute an advanced class of electrochemical energy storage devices that combine the advantageous characteristics of conventional capacitors and rechargeable batteries. By integrating high power-delivery capability with enhanced energy storage capacity, they provide an effective solution for applications requiring both rapid energy release and efficient energy storage. Unlike conventional batteries, where energy is predominantly stored through slower bulk redox reactions, SCs rely on either the reversible collection of charge at the electrode and electrolyte interface or rapid surface-controlled Faradaic processes. These mechanisms facilitate SCs to achieve high-power output, exceptionally rapid charging and discharging, and substantially improved cycling durability compared with many battery technologies. A conventional SC is composed of two electronically conductive and porous electrodes, an ion-conducting separator, and an electrolyte that facilitates ionic movement between the electrodes. Upon application of an external potential, ions within the electrolyte redistribute toward the oppositely charged electrode surfaces, where charge is stored through interfacial processes and/or fast redox reactions. When the device is discharged, the stored charge is released through the external circuit as the ions undergo redistribution within the electrolyte. The electrochemical characteristics of a supercapacitor are governed by more than a few interdependent aspects, which include the chemical and physical characteristics of the electrode materials, electrolyte category and composition, electrode morphology and structural configuration, pore-size distribution, and the kinetics of ion movement inside the electrode network.

The high-power characteristics of SCs arise primarily from the rapid kinetics of ionic and electronic charge transport and the surface-dominated nature of their energy storage processes. Since charge accumulation typically takes place at or close to the electrode-electrolyte interface and does not require extensive structural or phase changes within the active material, SCs can respond rapidly to high current demands. This surface-controlled mechanism also contributes to their outstanding durability, enabling repeated charging and discharging over very large numbers of cycles while maintaining relatively stable electrochemical performance. This combination of rapid response, high power output, and long cycling durability has positioned SCs as efficient energy storage technologies for diverse applications, including portable and flexible electronics, electric and hybrid vehicles, regenerative braking, grid-level power management, and wearable electronic systems.

### 2.2. Comparison Between Supercapacitors and Batteries

SCs and rechargeable batteries are the two extensively exploited electrochemical energy storage devices, both offering apparent advantages depending on the intended application. Although both devices store and deliver electrical energy, their charge storage mechanisms differ fundamentally. Batteries store energy through bulk electrochemical reactions involving ion insertion, conversion, or alloying processes within the electrode materials. These Faradaic reactions provide high specific energy but are generally accompanied by slower charge-transfer kinetics, resulting in relatively lower specific power and reduced cycle life. SCs employ two principal pathways for storing electrical energy. In EDLCs, energy is stored through the electrostatic separation of charges at the electrode–electrolyte interface. In pseudocapacitive systems, charge is instead stored through rapid, reversible Faradaic reactions resulting at or close to the surface of the electrode. Since the methods are predominantly interfacial and generally involve minimal structural rearrangement of the active material, SCs can undergo rapid charging and discharging while maintaining high-power output and outstanding extended cyclic durability.

**Figure 2:**
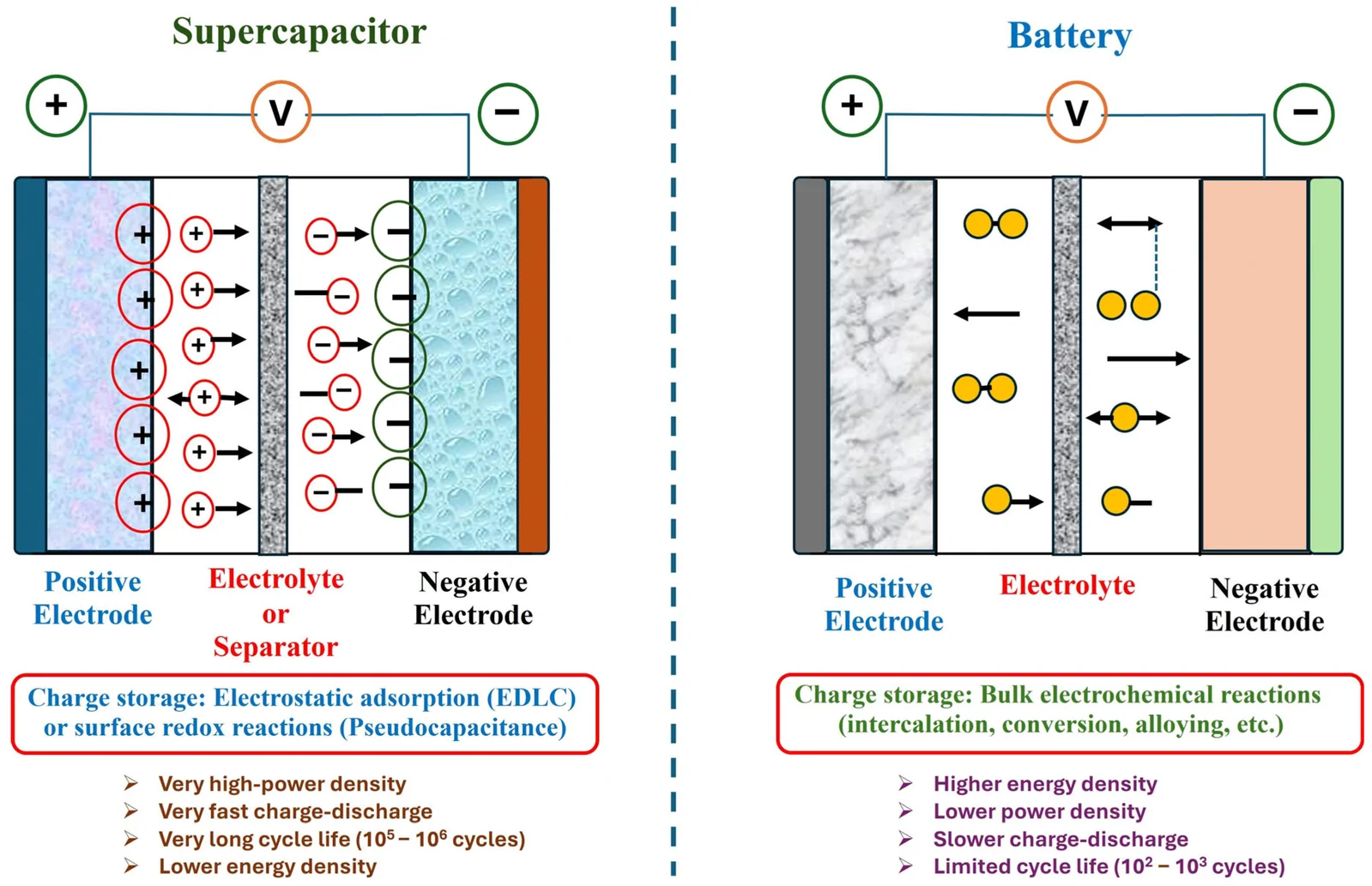
Schematic comparison between supercapacitors and rechargeable batteries, highlighting their mechanism of charge storage, electrochemical characteristics, and typical behavior metrics.

In practical applications, batteries are preferred where high energy storage and prolonged power delivery are required, such as in electric vehicles, portable electronics, and stationary energy storage systems. Conversely, SCs are particularly suitable for applications demanding rapid energy delivery or absorption over short durations, including regenerative braking, power backup systems, load leveling, wearable electronics, and pulse power devices. Consequently, SCs and batteries are progressively employed as matching technologies in hybrid energy storage systems, where the high specific energy of batteries is combined with the superior power capability and extensive cycle life of SC (Figure 2).

### 2.3. Ragone Plot: Relationship Between Energy Density and Power Density

**Figure 3:**
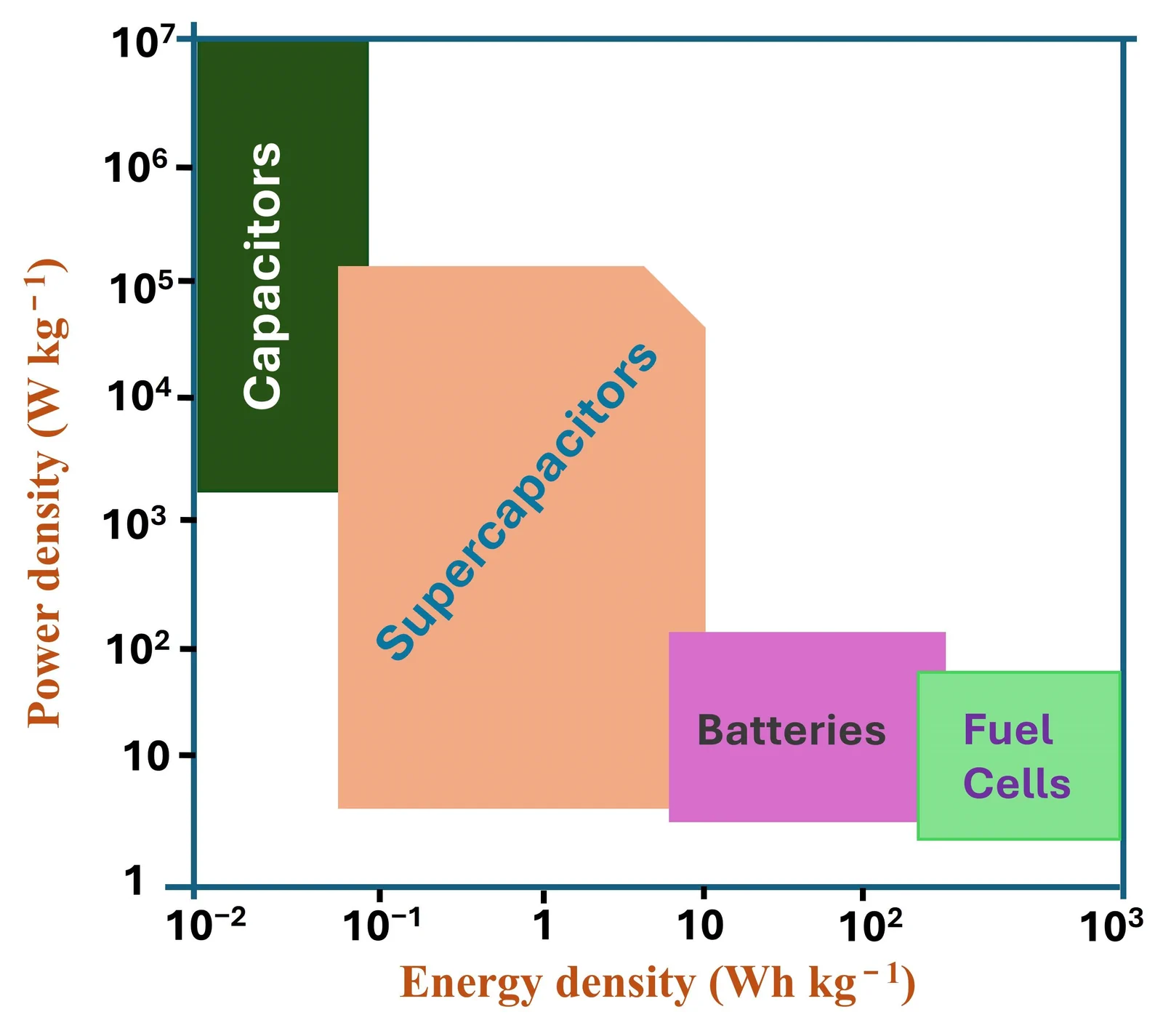
Typical Ragone plot illustrating the relationship between specific energy and specific power for conventional capacitors, SCs, hybrid capacitors, and rechargeable batteries.

The electrochemical behavior of energy storage systems is generally assessed by employing a Ragone plot, which illustrates the relationship between specific energy (Wh kg^−1^) and specific power (W kg^−1^) on logarithmic scales. This graphical representation provides a convenient means of comparing different energy storage technologies by simultaneously considering their energy storage capacity and power delivery capability. As illustrated in Figure 3, conventional dielectric capacitors occupy the region characterized by very high specific power but very low specific energy. Rechargeable batteries, particularly lithium-ion batteries, are positioned toward the high specific energy region, generally exhibiting comparatively lower specific power than conventional capacitors. Supercapacitors occupy an intermediate region, combining higher specific power than batteries with moderate specific energy. Hybrid capacitors aim to bridge this gap by combining battery-type and capacitor-type charge-storage characteristics.

The Ragone plot clearly demonstrates the intrinsic trade-off between specific energy and specific power. Systems capable of storing large amounts of energy generally deliver power more slowly, whereas those capable of supplying high power rapidly often possess lower energy storage capacity. Considerable research efforts have therefore focused on shifting the performance of supercapacitors toward the upper-right corner of the Ragone plot by simultaneously enhancing both specific energy and specific power.

### 2.4. Classification of Supercapacitors

**Figure 4:**
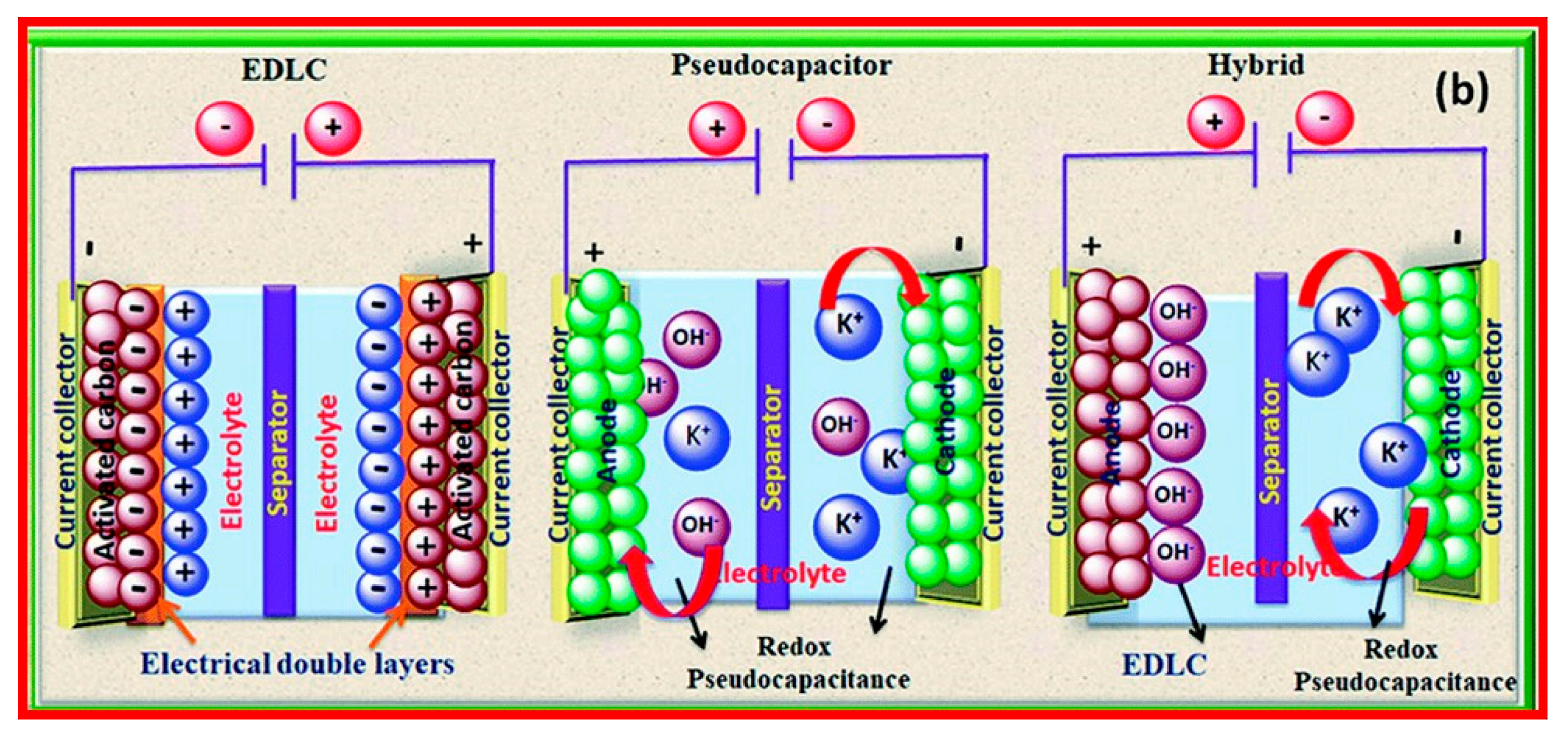
Types of supercapacitors along with their respective charge storage mechanisms. Reproduced with permission from [21].

SCs can be categorized into three principal types according to the underlying mechanism responsible for storing electric charge: EDLCs, PCs, and HSCs. A pictorial demonstration of the different types of supercapacitors is portrayed in Figure 4.

#### 2.4.1. Electrical Double-Layer Capacitors (EDLCs)

EDLCs store energy through electrostatic charge accumulation at the electrode–electrolyte interface, without involving Faradaic reactions [22,23,24,25,26,27]. When a potential is applied, electrolyte ions adsorb onto the surface of porous electrodes, forming an electrical double layer consisting of a charged electrode surface and an oppositely charged ionic layer in the electrolyte. Since no electron transfer or chemical transformation occurs during this process, EDLCs exhibit excellent reversibility, high specific power, and exceptional cyclic durability [28,29,30,31,32]. Carbon-derived materials are widely utilized as electrode components in EDLCs because of their high SSA, outstanding electrical conductivity, and chemical stability. A broad range of carbon-based structures, including activated carbon (AC), carbon nanotubes (CNTs), graphene, carbon nanofibers (CNFs), and various porous carbon architectures, have been extensively explored to enhance the electrochemical performance of EDLCs [33,34,35]. Though EDLCs possess outstanding power characteristics and long operational lifetimes, their energy densities are generally lower than those of battery systems because energy storage is limited to surface charge accumulation.

#### 2.4.2. Pseudocapacitors (PCs)

PCs accumulate and release charge through rapid and reversible Faradaic redox processes occurring at the electrode surface or within the near-surface region. Unlike EDLCs, pseudocapacitors involve electron transfer processes associated with oxidation–reduction reactions, electrosorption, or ion intercalation. These Faradaic processes contribute substantially to charge storage, resulting in higher capacitance and specific energy than purely electrostatic systems [36]. Transition metal oxides (TMOs), metal hydroxides, CPs, and metal sulfides are widely utilized as pseudocapacitive materials [37,38,39]. Typical examples include RuO_2_ [40,41,42], MnO_2_ [43,44,45], Ni(OH)_2_ [46], Co_3_O_4_ [47,48,49], polyaniline, polypyrrole, and PEDOT. Conducting polymers are attractive pseudocapacitive electrode materials because of their high theoretical capacitance, facile synthesis, and tunable electrical properties. However, repeated expansion and contraction during charge–discharge cycling often lead to structural damage and reduced long-term cycle life.

#### 2.4.3. Hybrid Supercapacitors (HSCs)

HSCs unite the advantages of both the EDLC and PSC materials to attain improved energy and power performance concurrently. These devices generally incorporate one battery-type or pseudocapacitive electrode and one capacitive electrode, thereby integrating multiple charge storage mechanisms within a single system [50,51,52,53,54,55]. Hybrid configurations can be categorized into asymmetric supercapacitors (ASCs), composite supercapacitors, and battery-supercapacitor hybrid systems. In ASCs, various electrode materials are employed for the anode and cathode to expand the working voltage range and improve the specific energy. Composite electrodes consisting of carbon-derived materials combined with CPs and TMOs are also widely investigated because they offer synergistic improvements in conductivity, capacitance, and cycling stability. HSCs are an attractive, efficient candidate for next-generation energy storage systems because they effectively bridge the performance gap between conventional capacitors and batteries. Table 1 offers a complete assessment of the major types of supercapacitors, highlighting their typical electrode materials, charge storage mechanisms, and key performance characteristics.

## 3. Key Performance Parameters

The performance of supercapacitors is commonly evaluated using several electrochemical parameters that provide insights into their energy storage capability and operational efficiency.

### 3.1. Specific Capacitance and Specific Capacity

Specific capacitance is a key electrochemical metric for evaluating the charge-storage performance of electrode materials. It describes the quantity of electrical charge that can be stored by the active electrode material relative to its mass, surface area, or volume during the charging process. Depending on the nature of the electrode and the method used for electrochemical characterization, capacitance can be reported in different forms, namely gravimetric capacitance (F g^−1^), areal capacitance (F cm^−2^), or volumetric capacitance (F cm^−3^). Among these, gravimetric capacitance is the most widely used parameter for evaluating EDLC-based electrode materials because it enables straightforward comparison of electrochemical performance across studies. In contrast, the electrochemical performance of pseudocapacitive and battery-type electrode materials is increasingly reported in terms of specific capacity, expressed in coulombs per gram (C g^−1^) or milliampere hours per gram (mAh g^−1^), because these units more accurately represent Faradaic charge-storage behavior. Therefore, it is essential to distinguish between capacitance- and capacity-based metrics when comparing the electrochemical behavior of different classes of supercapacitor electrodes.

### 3.2. Energy Density

Energy density represents the quantity of electrical energy that can be stored by a device relative to its mass or volume and is commonly reported in Wh kg^−1^ or Wh L^−1^. This parameter indicates how long a SC can continuously supply energy to an external load. The attainable energy density is primarily governed by electrode capacitance and the voltage range over which the device can operate. Although supercapacitors generally provide lower energy storage capacity than conventional batteries, substantial progress has been made in enhancing their energy density through the design of high-performance electrode materials and the integration of ASC architectures.

### 3.3. Power Density

Power density represents the rate at which energy can be delivered and is generally expressed in W kg^−1^. One of the distinguishing features of SCs is their extremely high power density, enabling fast charging–discharging within seconds or minutes. High electrical conductivity, efficient ionic transport, and low internal resistance are crucial for accomplishing superior power performance.

### 3.4. Cycling Stability

Cycling stability describes the capability of a supercapacitor to withstand its electrochemical behavior during repeated charge and discharge operations. It is typically evaluated by monitoring capacitance retention over thousands or even hundreds of thousands of cycles. Excellent cycling stability is vital for real-world applications because it directly affects device lifetime and reliability. Carbon-based EDLCs often exhibit capacitance retentions exceeding 95% after extensive cycling, whereas conducting polymer electrodes generally experience gradual degradation due to structural changes during repeated redox processes.

Cycling stability should be interpreted in conjunction with the testing conditions rather than by considering the number of charge–discharge cycles alone. Reported capacitance retention depends on the applied current density or scan rate, potential window, electrolyte, electrode mass loading, and whether the measurement was performed using a three-electrode or two-electrode configuration. Therefore, direct durability comparisons among different polymer/graphene composite electrodes should be made cautiously when the testing conditions differ substantially. Where available, the cycling current density, device configuration, and coulombic efficiency should be considered together with the reported capacitance retention. In addition, an increase in capacitance during the initial cycles may arise from electrode activation, enhanced electrolyte penetration, or gradual wetting of electrochemically accessible sites and should not automatically be interpreted as superior long-term stability. Accordingly, capacitance retention in this review is reported relative to the appropriate initial or stabilized capacitance value, as provided by the original study, and long-term decay is distinguished from initial activation wherever the reported data permits. Capacitance retention is generally calculated as the ratio of the capacitance after a specified number of charge–discharge cycles to the initial capacitance, expressed as a percentage: C_N_/C_0_ × 100, where C_0_ is the initial capacitance, and C_N_ is the capacitance after N cycles. However, when the original study defines the retention relative to a stabilized capacitance or another reference point, the reported definition is retained for consistency with the source.

### 3.5. Mass Balancing for Asymmetric Devices

Mass balancing is an essential consideration in the design and evaluation of asymmetric supercapacitor (ASC) devices, in which the positive and negative electrodes may have different charge-storage characteristics and potential windows. To ensure effective utilization of both electrodes, the charges stored by the positive and negative electrodes should be appropriately balanced according to the charge-balance condition, q^+^ = q^−^. For capacitance-based electrodes, the charge balance can be expressed as follows:

m^+^C^+^ΔV^+^ = m^−^C^−^ΔV^−^

and the corresponding mass ratio is therefore given by the following:

$${\frac{m}{m^{-}}}^{+}=\frac{C^{-}{\Delta V}^{-}}{C^{+}{\Delta V}^{+}}$$

where m^+^ and m^−^ are the active masses of the positive and negative electrodes, respectively; C^+^ and C^−^ are their corresponding specific capacitances; and ΔV^+^ and ΔV^−^ represent their respective potential windows. The specific capacitances and potential windows should preferably be determined from the corresponding three-electrode measurements before assembling the asymmetric device. Appropriate mass balancing helps maximize the usable cell voltage and active-material utilization while minimizing electrode polarization, overcharging, and undesirable side reactions. For battery-type or Faradaic electrodes for which charge storage is more appropriately expressed in terms of specific capacity rather than capacitance, the charge-balance relationship should instead be formulated using the corresponding specific capacities (q^+^ and q^−^). Thus, the mass ratio should be determined according to the charge-storage mechanism of the individual electrodes rather than applying a single capacitance-based expression to all asymmetric systems. The positive-to-negative mass ratio and the corresponding mass basis should be clearly reported when evaluating the performance of asymmetric devices.

### 3.6. Coulombic Efficiency

Coulombic efficiency is defined as the ratio of the discharge to the charge supplied during charging and is usually expressed as a percentage (%). This parameter reflects the reversibility and efficiency of charge storage processes within the device. High coulombic efficiency indicates minimal energy loss during operation and efficient utilization of stored charge. Values approaching 100% are generally desirable for practical supercapacitor applications.

The electrochemical equations usually employed to estimate the supercapacitor behavior of the electrode materials and devices, together with their corresponding units, are summarized in Table 2. These equations provide a standardized framework for calculating main performance factors, which include specific capacitance, specific capacity, energy density, power density, coulombic efficiency, and electrode mass balance.

## 4. Polymer and Graphene-Based Composites for Supercapacitors

Polymeric materials have attracted substantial consideration in supercapacitor research due to their structural flexibility, ease of processing, and adjustable physico-chemical characteristics. Various polymers, including polyvinylidene fluoride (PVDF), polytetrafluoroethylene (PTFE), conducting polymers such as PANI, PPy, PTh, PEDOT, and HCPs [56,57], have been investigated for supercapacitor applications. Nevertheless, each class of polymer exhibits inherent limitations that restrict its practical electrochemical performance. PVDF and PTFE are electrically insulating polymers and are therefore primarily employed as binder materials rather than active electrode materials. In addition, their hydrophobic nature may reduce electrolyte wettability, thereby limiting efficient ion transport within the electrode. HCPs possess exceptionally high surface areas and well-developed porous structures; however, their intrinsically low electrical conductivity limits their direct use as supercapacitor electrodes. Consequently, HCPs are often subjected to carbonization or pyrolysis to produce conductive porous carbon materials with enhanced electrochemical performance. In contrast, conducting polymers (PANI, PPy, PTh, and PEDOT) are electrochemically active materials capable of delivering high pseudocapacitance via fast and reversible redox reactions. Despite these merits, their practical application is hindered by poor long-term cycling stability caused by repeated volumetric enlargement and shrinkage during doping and de-doping processes. These structural changes frequently induce mechanical stress, cracking, delamination, and degradation of the polymer framework, resulting in progressive capacitance loss during repeated discharge cycles. Furthermore, CPs often suffer from limited mechanical robustness, relatively low-rate capability at high current densities, and poor structural stability during long-term operation.

To overcome these obstacles, wide-ranging research has focused on developing polymer-based composite electrodes by integrating conducting polymers with highly conductive nanomaterials such as graphene, graphene derivatives, CNTs, MXenes, metal oxides, metal sulfides, and other nanostructured materials. Among the various reinforcing materials investigated, graphene has attracted considerable attention as a highly effective candidate due to its outstanding electrical conductivity, exceptionally large theoretical SSA, superior mechanical robustness, and excellent chemical stability. The inclusion of graphene into polymer matrices may help accommodate polymer volume changes and may contribute to improved electronic connectivity, electrolyte accessibility, and structural stability. The extent of these effects depends on the composite architecture and should be assessed using appropriate structural, electrochemical, and physicochemical characterization. Consequently, polymer/graphene-based composites have become one of the most thoroughly explored classes of electrode materials for next-generation high-performance SCs. The following sections discuss recent advances in polymer/graphene composites, highlighting their synthesis strategies, electrochemical performance, and the underlying mechanisms responsible for their enhanced energy storage properties.

### 4.1. Important Characteristics of Graphene-Derived Materials for SC Applications

The electrochemical performance of polymer/graphene composite electrodes is strongly influenced by the physicochemical properties of graphene-derived materials. Although graphene possesses high theoretical electrical conductivity and a large specific surface area, its actual contribution depends on the accessible surface area, pore structure, chemical composition, electrical connectivity, wettability, and interaction with the polymer matrix. Therefore, graphene selection should consider multiple structural and interfacial properties rather than specific surface area alone [58].

Chemical composition and surface functional groups: GO contains hydroxyl, epoxy, carbonyl, and carboxyl groups that improve hydrophilicity, dispersion, and interaction with polymer chains. These groups enhance adhesion and reduce graphene restacking. However, excessive oxidation disrupts the *sp^2^* carbon network and decreases electrical conductivity. Surface functionalization must therefore be balanced against conductivity [59].

Degree of reduction and oxygen content: Reduction restores part of the *sp^2^* carbon network and improves electrical conductivity but may reduce hydrophilicity, dispersion, and polymer interaction. Highly oxidized graphene offers better dispersibility but lower conductivity. Thus, an optimum oxidation state is required to balance conductivity, wettability, and electrochemical accessibility [60].

Defect density and edge-site concentration: Defects and edge sites can provide additional ion adsorption sites and improve polymer interactions. However, excessive defects interrupt the carbon network and increase charge-transfer resistance. Controlled defect engineering is therefore preferred [61].

Lateral size, thickness, and number of graphene layers: Graphene nanosheets with suitable lateral dimensions can improve interfacial contact and conductive network formation. Extremely small fragments may contain excessive defects, whereas large nanosheets and multilayer structures can restack and restrict ion transport. Therefore, graphene dimensions should be optimized according to the polymer structure and electrolyte properties [62].

Specific surface area: SSA influences the area available for electrical double layer formation. However, BET surface area does not always represent the electrochemically accessible surface because pores may be blocked by polymer chains or graphene restacking [63].

Pore architecture and pore-size distribution: Hierarchical pores containing micropores, mesopores, and macropores can improve charge storage and ion transport. Micropores support charge accumulation, mesopores facilitate ion diffusion, and macropores improve electrolyte penetration. However, pore accessibility depends on electrolyte ion size, electrode thickness, and pore dimensions [64].

Electrical conductivity and network connectivity: A continuous graphene network improves electron transport and reduces internal resistance. Nevertheless, electrode conductivity also depends on graphene loading, dispersion, contact resistance, polymer coverage, and restacking. Excessive graphene content may reduce the proportion of electroactive polymers. Therefore, graphene loading should be optimized to achieve effective conductivity without diluting the active material [65].

Surface wettability and dispersibility: Good wettability facilitates electrolyte penetration and ion contact with active surfaces, while uniform dispersion prevents aggregation and uneven polymer deposition. GO generally provides better hydrophilicity than rGO. Functionalization, in situ polymerization, and controlled solution blending may improve dispersion without significantly compromising conductivity [66].

### 4.2. Polymer/Graphene Composites

Choosing a suitable polymer matrix is vital for maximizing the electrochemical behavior of graphene-reinforced composites. The polymer should possess desirable characteristics such as high electrical conductivity, excellent mechanical robustness, thermal stability, and good processability. In addition, it should facilitate homogeneous graphene dispersion, promote strong interfacial bonding, and maintain the structural stability of the resulting composite [67]. PVDF is a fluoropolymer widely recognized for its excellent chemical resistance, mechanical durability, thermal stability, and long-term environmental stability. Although PVDF itself is electrochemically inactive and electrically insulating, it plays an indispensable role in graphene-based supercapacitor electrodes as a polymeric binder [68,69]. Its primary function is to firmly anchor graphene sheets or other active materials onto the current collector, thereby preserving electrode integrity and providing sufficient mechanical strength during repeated charge–discharge cycles. Typically, graphene-based electrodes are prepared by dispersing graphene with approximately 10–20 wt% PVDF in N-methyl-2-pyrrolidone (NMP) or dimethylformamide (DMF) to attain a homogeneous thick slurry. The thick slurry is subsequently deposited onto conductive substrates using conventional techniques including doctor-blade coating, spray coating, or drop casting, followed by drying and compression to obtain robust electrodes. The amount of PVDF must be carefully optimized because insufficient binder results in poor adhesion between the active material and the current collector, whereas excessive PVDF increases the insulating content of the electrode, thereby hindering electron transport and reducing both specific capacitance and rate capability. To compensate for this drawback, conductive carbon additives such as carbon black or acetylene black are frequently incorporated into the electrode formulation [70,71]. These conductive additives improve electrical conductivity, minimize graphene sheet aggregation, facilitate electron transport pathways, and enhance cycling stability without significantly altering the electrode morphology. Besides conventional slurry coating, vacuum filtration has also emerged as an effective approach for fabricating graphene/PVDF composite electrodes, particularly on porous current collectors such as nickel foam [72]. This technique enables uniform deposition of graphene while allowing precise control over the loading and distribution of active materials through filtration parameters. Electrodes prepared by vacuum filtration generally exhibit excellent structural uniformity, good mechanical stability, high specific capacitance, and outstanding capacitance retention during prolonged electrochemical cycling.

For instance, Jinyang Dong et al. [73] used a graphene/PVDF composite binder to improve the electrochemical behavior of MnO_2_-derived supercapacitor electrodes. When compared to the conventional PVDF binder, the graphene/PVDF composite binder achieved a higher specific capacitance of 220 F g^−1^ at 0.5 A g^−1^, with an optimum graphene loading of 5 wt%. The composite electrode also delivered outstanding durability, maintaining 90% of its original specific capacitance over 1000 GCD cycles. The improved electrochemical behavior was attributed to the strong interface between graphene and PVDF, which created effective electron transport pathways, reduced charge-transfer resistance, and improved electrical connectivity between MnO_2_ particles. Pazhamalai et al. reported a free-standing PVDF/rGO composite film as a flexible electrode for all-solid-state supercapacitors [74]. The inclusion of rGO into the PVDF matrix substantially improved the electrical conductivity and charge storage capability of the composite, delivering a supreme specific capacitance of 683 mF cm^−2^ at 10 μA cm^−2^. The device also showed exceptional rate capability, with a coulombic efficiency exceeding 80%, and sustained ~87% of its original specific capacitance over 10,000 charging and discharging cycles, representing its potential for flexible energy storage applications.

PTFE is another fluoropolymer that has been extensively explored as a binder material for graphene- and carbon-based supercapacitor electrodes. Unlike PVDF, PTFE is commercially available as an aqueous dispersion or alcohol-based suspension, eliminating the need for hazardous organic solvents during electrode fabrication [75,76,77,78]. This feature has made PTFE particularly attractive for mass production due to its lower processing cost, improved safety, and environmentally friendly manufacturing. Few research findings have supported that the selection of binder predominantly impacts the structural and electrochemical properties of supercapacitor electrodes [79,80]. Comparative investigations between PTFE and PVDF-based electrodes have shown that they generally promote a more porous electrode architecture with a large microporous volume, facilitating electrolyte ion diffusion and resulting in higher specific capacitance. These findings indicate that binder selection affects not only the mechanical stability of the electrode but also its pore structure and charge-storage behavior. Despite these advantages, PTFE is electrically insulating and highly hydrophobic, which can adversely affect electrode performance when used in excessive amounts. Increased PTFE content may reduce electrical conductivity and restrict electrolyte penetration into the porous electrode network, thereby decreasing specific capacitance, energy density, and rate capability. Conversely, insufficient binder loading weakens the adhesion between active materials and the current collector, leading to poor mechanical stability and reduced cycling durability. Accordingly, several researchers have reported that the optimum PTFE content depends on factors such as the active material, current collector, and electrolyte composition, with typical binder concentrations ranging from approximately 5 to 10 wt%. To overcome the inherent hydrophobicity of PTFE, researchers have introduced hydrophilic additives such as polyvinylpyrrolidone (PVP). Previous studies demonstrated that incorporating a small amount of PVP substantially improves electrode wettability by reducing the water contact angle, thereby enhancing electrolyte accessibility to the porous electrode structure and improving electrochemical performance [81,82]. The electrochemical behavior of PTFE-based graphene electrodes has also been reported to depend strongly on the electrolyte employed. Comparative studies using aqueous and organic electrolytes revealed that aqueous alkaline electrolytes generally provide higher specific capacitance because of their superior conductivity and more effective ionic transport [83]. In contrast, organic electrolytes typically exhibit lower capacitance but offer a wider operating voltage range, which is favorable for achieving higher specific energy. These findings emphasize that the overall performance of graphene/PTFE supercapacitors is governed by the combined effects of the binder, electrolyte, current collector, and active material, highlighting the importance of optimizing all electrode components simultaneously.

### 4.3. Conducting Polymers/Graphene Composites

**Figure 5:**
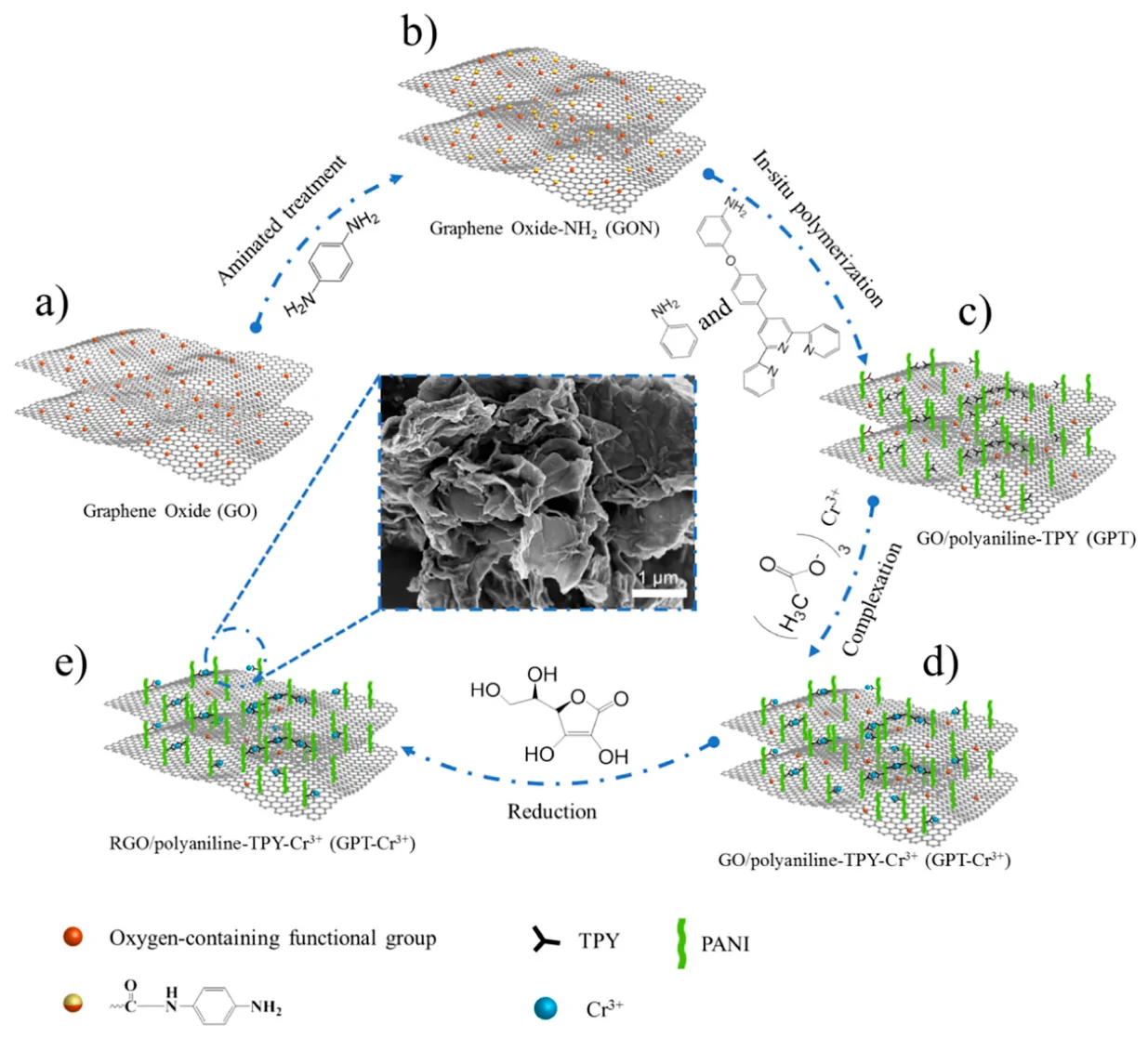
Pictorial representation of the composite preparation involving graphene derivatives and polymers involving in situ polymerization. Reproduced with permission from [88].

rGO is frequently incorporated into conducting polymers to overcome the intrinsic demerits of weak cycling durability and limited electrical conductivity. Owing to its outstanding electrical conductivity, high SSA, and superb mechanical strength, rGO offers efficient electron transport pathways while suppressing CPs volume enlargement during repeated charging and discharging cycles. Consequently, rGO/conducting polymer composites generally exhibit greater capacitance, enhanced rate capability, and improved cycling durability than pristine CPs. For the fabrication of CP/graphene-derived composites intended for SC applications, a widely adopted approach involves the in situ polymerization of the conducting polymer directly onto graphene-based sheets. The process is generally carried out at ambient temperature for several hours under continuous mechanical stirring, as illustrated in Figure 5 [84,85,86,87,88].

### 4.4. PANI/rGO

**Figure 6:**
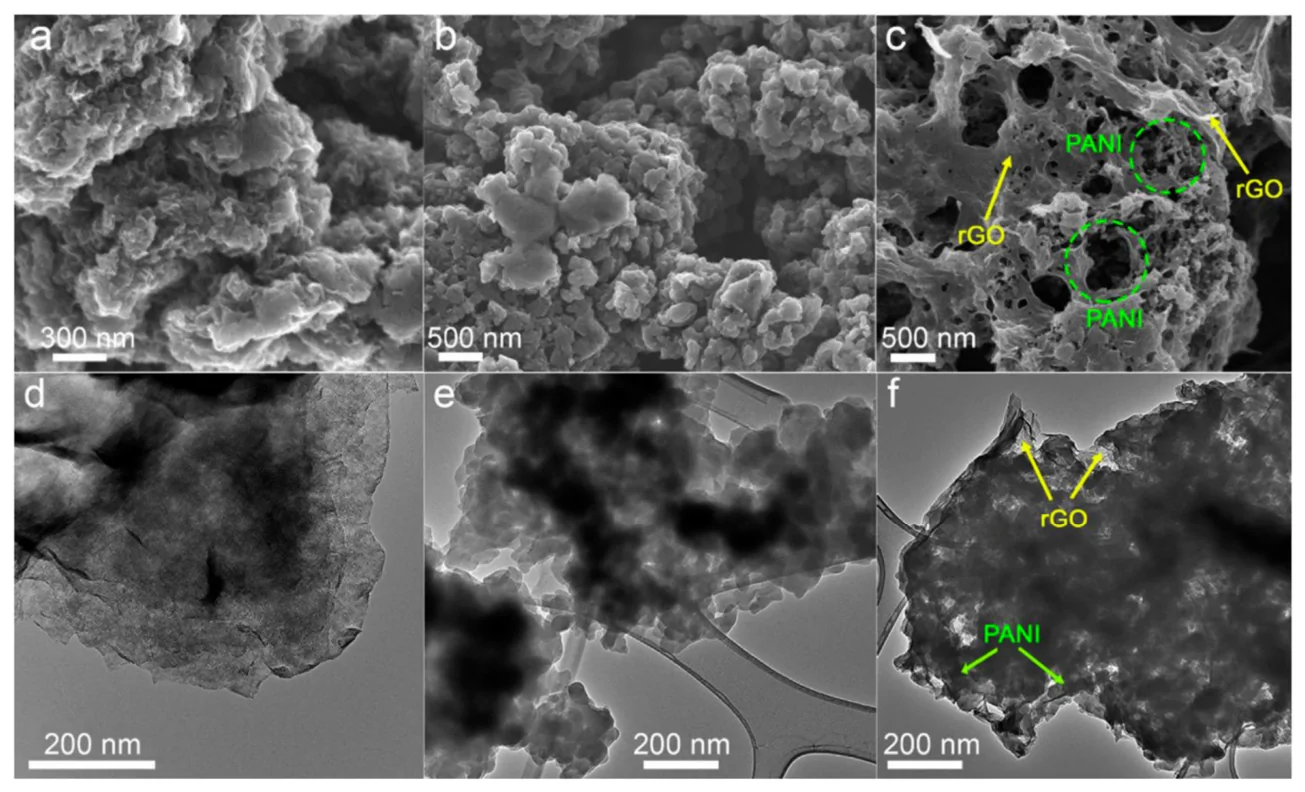
FE-SEM and HR-TEM micrographs of rGO-HT (**a**,**d**), PANI (**b**,**e**), and the PANI/rGO-HT composite (**c**,**f**). In the composite images, PANI and rGO are highlighted in green and yellow, respectively. Reproduced with permission from [89].

Moyseowicz et al. developed a porous polyaniline/rGO composite via a facile dual-step approach employing the oxidative polymerization of aniline (OPA) and subsequent hydrothermal treatment of PANI with GO [89]. The hydrothermal process reduced GO and simultaneously generated a three-dimensional interconnected porous framework, in which PANI nanoparticles were uniformly anchored onto the rGO nanosheets. FE-SEM and HR-TEM images of rGO, PANI, and the PANI/rGO composite are depicted in Figure 6a–f. The rGO exhibits typical crumpled and agglomerated nanosheets (Figure 6a), while pristine PANI forms densely packed, coral-like nanoparticle aggregates (Figure 6b). In contrast, the PANI/rGO composite displays a three-dimensional porous architecture in which interconnected rGO nanosheets uniformly support PANI nanoparticles (Figure 6c). Strong interfacial interactions, including π–π stacking, hydrogen bonding, and electrostatic attraction, promote the uniform distribution of PANI on the graphene surface. Hydrothermal treatment also generates an interconnected porous framework, which is beneficial for electrolyte penetration and ion diffusion. HR-TEM images further reveal the thin, wrinkled morphology of rGO sheets (Figure 6d) and the granular morphology of PANI aggregates (Figure 6e). In the composite, PANI nanoparticles are homogeneously adhered to the rGO nanosheets (Figure 6f), forming a well-connected conductive network that improves electronic transport and provides abundant electrochemically active sites for high-performance supercapacitors. The resulting composite exhibited an SSA of 228 m^2^ g^−1^. This surface area, together with the observed composite morphology, may contribute to the availability of charge-storage sites and electrolyte-ion accessibility. However, the electrochemically accessible surface area and ion-transport behavior cannot be established from the specific surface area alone. Owing to the synergistic blend of the high pseudocapacitance of PANI and the excellent electrical conductivity and structural integrity of rGO, the PANI/rGO-HT electrode delivered a specific capacitance of 420 F g^−1^ at 0.2 A g^−1^, while retaining 239 F g^−1^ at a high current density of 20 A g^−1^, demonstrating excellent rate capability. Moreover, the electrode exhibited remarkable cycling durability with 80% capacitance maintenance over 6000 charge–discharge cycles, which was significantly superior to pristine PANI. Additionally, the constructed symmetric supercapacitor (SSC) achieved a specific capacitance of 220 F g^−1^, a maximum specific energy of 9.3 Wh kg^−1^ at a specific power of 0.1 kW kg^−1,^ and maintained a specific energy of 4.4 Wh kg^−1^ even at a high specific power of 11.6 kW kg^−1^. The outstanding electrochemical behavior was ascribed to the 3D porous architecture, which enhanced electrolyte accessibility, facilitated quick ionic and electronic transport pathways, and effectively mitigated the structural degradation of PANI during charge–discharge cycling, making the composite a potential candidate for high-performance SCs.

Surfactants are widely employed during the synthesis of conducting polymer composites to regulate nucleation, suppress graphene aggregation, and tailor the morphology of PANI. Among them, cetyltrimethylammonium bromide (CTAB) acts as a soft template that promotes the formation of uniform PANI nanofibers while concurrently improving graphene dispersion. This results in increased electrochemically active surface area, faster ion diffusion, and improved electron transport. Samada et al. systematically investigated different CTAB concentrations (0.04 to 0.10 M) during PANI/rGO synthesis and found that 0.08 M CTAB produced uniformly distributed PANI nanofibers (50–80 nm) [90]. The prepared electrode exhibited 704 F g^−1^, outstanding rate capability, 23.3 Wh kg^−1^, 850 W kg^−1^, and retained 86% capacitance over 5000 cycles, demonstrating the critical role of surfactant-assisted morphology control.

Although carbon-based materials and CPs have frequently been combined to form binary composites, increasing research interest has recently shifted toward multicomponent systems containing three distinct phases. The incorporation of complementary components into a single composite can create synergistic interactions, thereby overcoming some of the individual limitations of the constituent materials and improving the overall electrochemical properties [91,92]. In particular, ternary architectures composed of PANI, carbonaceous materials, and metal-containing compounds have been developed as efficient electrode materials for electrochemical energy storage utilization [93,94,95,96]. The integration of these three components can provide high electrical conductivity, ample electrochemically accessible sites, favorable surface characteristics, and improved structural integrity. Consequently, PANI/carbon/metal-based ternary composites have been reported to exhibit enhanced specific capacitance, greater resistance to chemical degradation, improved nanostructural features, and better charge–discharge durability than many corresponding single- or two-component systems [97,98]. These synergistic characteristics, together with their potential for relatively cost-effective fabrication and application in high-performance energy storage technologies, have stimulated considerable interest in the progress of ternary nanocomposites as advanced electrode materials for SCs [99,100,101,102].

Although PANI/rGO composites possess excellent electrical conductivity and pseudocapacitive behavior, their capacitance is still restricted by the limited number of redox-active sites. The incorporation of transition-metal oxides may introduce additional Faradaic charge-storage contributions and may influence the electrochemically accessible surface area and structural stability of PANI/rGO-based composites. The extent to which these factors contribute to the observed electrochemical performance depends on the composition and structure of the composite and should be supported by appropriate electrochemical and physicochemical characterizations. MnO_2_ is among the most attractive pseudocapacitance materials owing to its high theoretical capacitance, cost-effectiveness, eco-friendliness, and variable valence states. Do Thi Thuy et al. assembled a 3D-printed rGO/PANI/MnO_2_ ternary electrode material followed by electrodeposition of MnO_2_ nanoparticles [103]. The optimized electrode delivered a specific capacitance of 740 F g^−1^ at 1 A g^−1^ and maintained 97% of its initial capacitance over 5000 cycles, demonstrating that MnO_2_ significantly improves cycling durability while contributing additional Faradaic charge storage.

**Figure 7:**
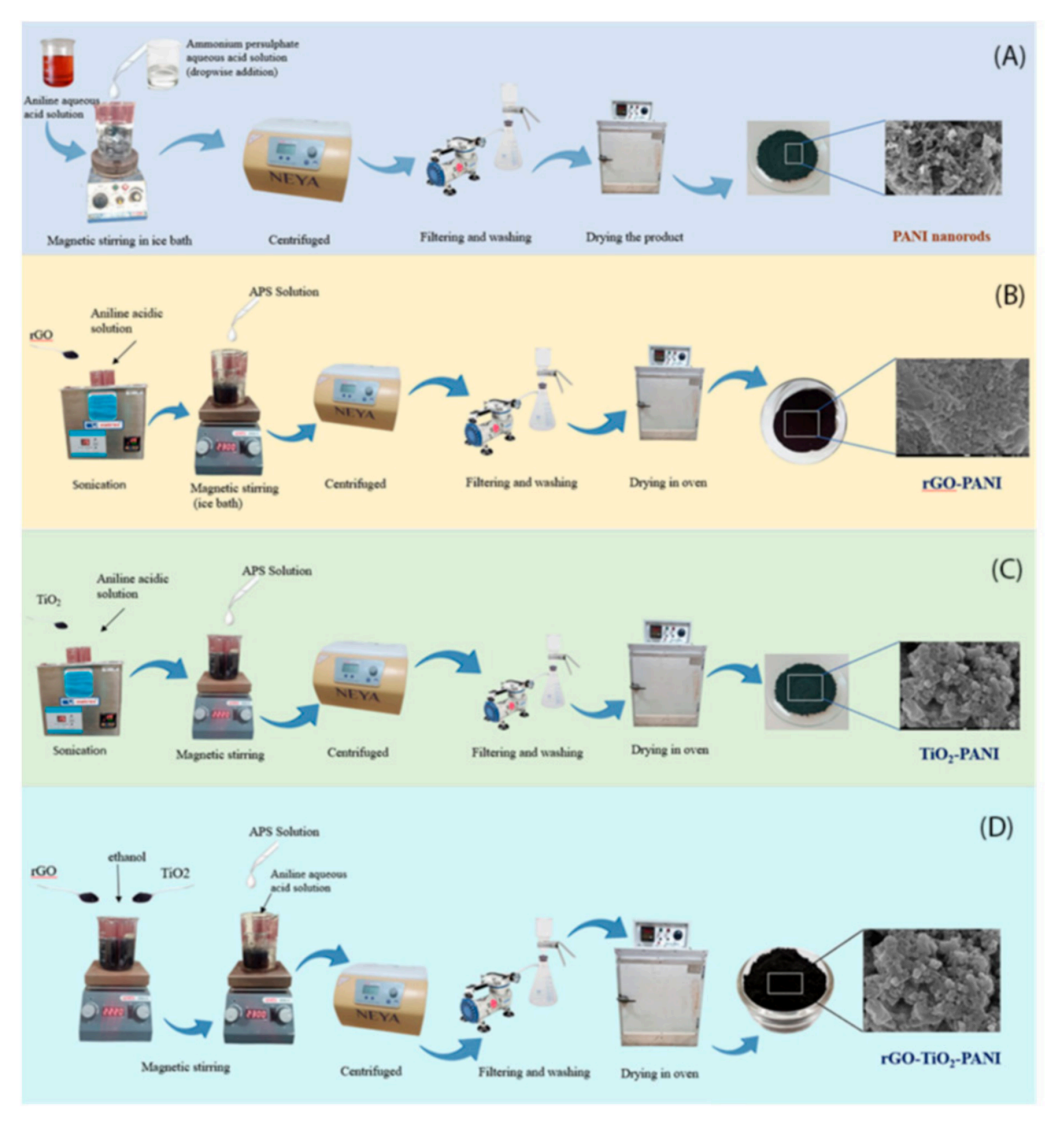
Pictorial representation of the synthesis procedures for (**A**) PANI, (**B**) rGO-PANI, (**C**) TiO_2_-PANI, and (**D**) rGO-TiO_2_-PANI composites prepared via chemical oxidative polymerization. Reproduced with permission from [104].

TiO_2_ is commonly incorporated into conducting polymer composites because of its excellent chemical stability, mechanical robustness, and ability to suppress the structural degradation of PANI during repeated cycling. Furthermore, TiO_2_ improves electrode integrity and enhances electrolyte accessibility. Bhawna et al. synthesized an interconnected rGO-TiO_2_-PANI ternary composite through in situ OPA [104]. The fabrication routes for PANI, rGO-PANI, TiO_2_-PANI, and the ternary rGO-TiO_2_-PANI composites are schematically illustrated in Figure 7. Pristine PANI was synthesized through conventional chemical OPA, where aniline was polymerized in an acidic medium using ammonium persulfate (APS) as the oxidizing agent, followed by purification and drying (Figure 7A), ensuring uniform growth of PANI on the graphene sheets (Figure 7B). Similarly, the TiO_2_-PANI composite was attained through OPA in the presence of TiO_2_ nanoparticles, resulting in intimate polymer–oxide interactions (Figure 7C). For the ternary rGO-TiO_2_-PANI composite, rGO and TiO_2_ were initially dispersed to form a hybrid support, followed by in situ OPA employing APS to produce a uniformly integrated ternary nanocomposite (Figure 7D). After synthesis, all products were purified by repeated washing, separated by centrifugation or filtration, and dried to obtain the final composite powders. The corresponding FE-SEM images of each synthesized material are presented alongside the respective synthesis routes in Figure 7, providing direct insight into their morphological characteristics. FE-SEM pictures disclose definite morphological variations among the prepared samples, confirming the successful absorption of rGO and TiO_2_ into the PANI matrix. Pristine PANI exhibits a dense network of interconnected nanorods with reasonably even surfaces. In contrast, the rGO-PANI composite displays crumpled, layered sheets with wrinkled and rough surfaces, indicating the uniform incorporation of rGO and an increased surface area. The TiO_2_-PANI composite consists of finely dispersed TiO_2_ nanoparticles embedded within the PANI matrix, forming a porous and rough architecture. The ternary rGO-TiO_2_-PANI composite exhibits a highly interconnected, porous network with folded and fractured features, demonstrating the effective integration of both rGO and TiO_2_ into PANI and the formation of an open conductive framework favorable for electrochemical applications. The composite exhibited a reported specific capacitance of 4733.55 F g^−1^ and a capacitance retention of 89.9% after 3000 cycles. However, the reported specific capacitance of 4733.55 F g^−1^ was calculated from CV measurements at a scan rate of 3 mV s^−1^ using a three-electrode configuration. In comparison, the specific capacitance calculated from GCD measurements was 252.52 F g^−1^ at a current density of 0.5 A g^−1^. This substantial discrepancy highlights the importance of considering the measurement technique, experimental conditions, and calculation procedure when evaluating the reported capacitance. Furthermore, the three-electrode configuration should be taken into account when comparing these results with those obtained from complete two-electrode devices. The unusually high CV-derived capacitance should therefore be interpreted cautiously and should not be considered directly equivalent to the GCD-derived capacitance or the performance of a complete supercapacitor device.

Lead oxide (PbO_2_) possesses excellent electrical conductivity, multiple oxidation states, and high electrical activity, making it an efficient pseudocapacitive material for HSC electrodes. Akyol et al. prepared PANI/Cl-GO/PbO_2_ ternary electrodes using chemically synthesized PANI and chlorine-doped graphene oxide combined with PbO_2_ [105]. The ASC system exhibited an areal capacitance of 97 mF cm^−2^ in acidic electrolyte and 54 mF cm^−2^ in alkaline electrolyte, highlighting the influence of electrolyte chemistry on the electrochemical behavior of PbO_2_-containing hybrid electrodes.

### 4.5. PPy/rGO

PPy is one of the most extensively studied polymers for supercapacitor electrodes owing to its high electrical conductivity, rapid and reversible redox activity, simple synthesis, and environmental stability. Nevertheless, pristine PPy suffers from poor mechanical integrity and rapid capacitance fading caused by repeated swelling and shrinkage during continuous charge–discharge cycling. The incorporation of rGO may help mitigate some limitations of PPy by providing a conductive framework and influencing the structural characteristics of the composite. Depending on the composite architecture, rGO may contribute to improved electronic connectivity and electrolyte accessibility. Its influence on polymer volume changes and graphene restacking, however, depends on the material’s structure and should be confirmed through appropriate structural and electrochemical characterization.

**Figure 8:**
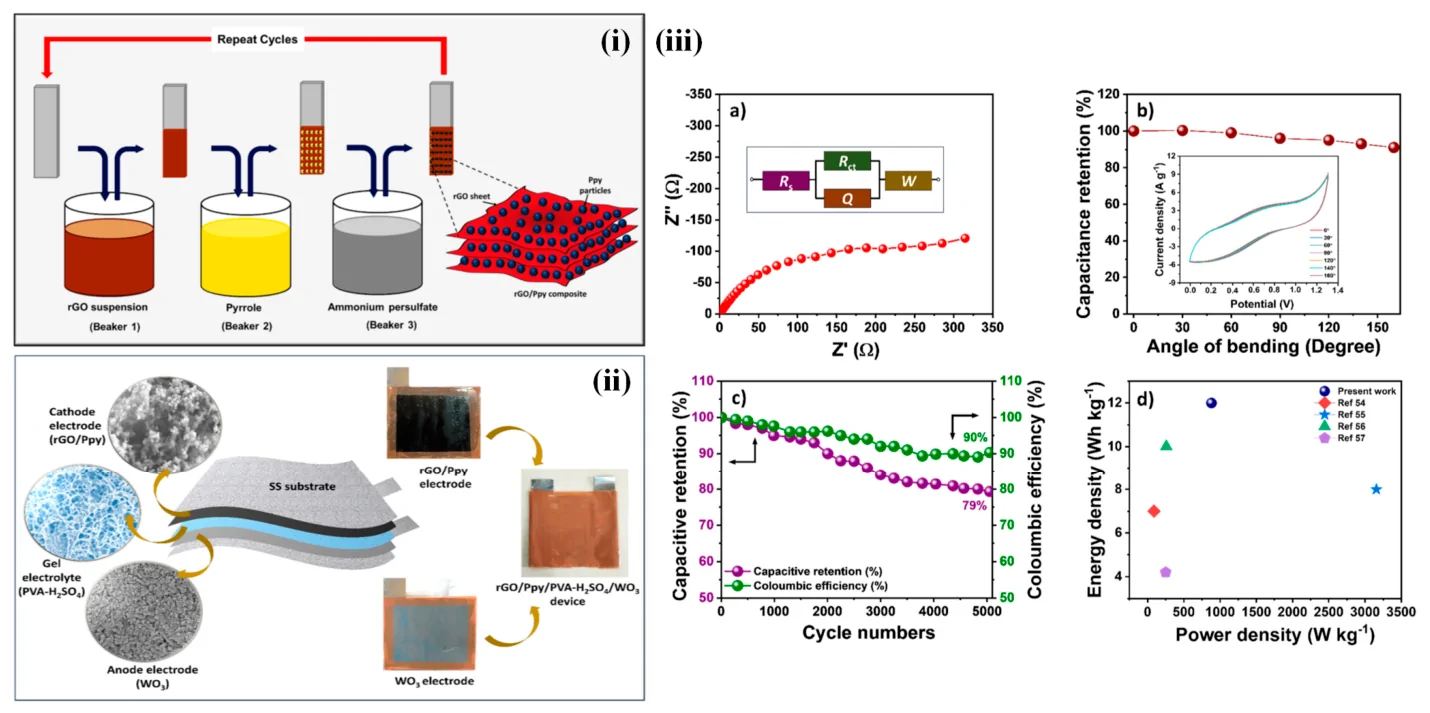
(**i**) Graphical representation of the SILAR-assisted fabrication of PPy/rGO composite thin films on a SS substrate, (**ii**) schematic presentation of the fabrication and assembly of the FSSC employing an PPy/rGO cathode, WO_3_ anode, and PVA-H_2_SO_4_ gel electrolyte, (**iii**) electrochemical and mechanical performance of the device: (**a**) Nyquist plot with the corresponding equivalent circuit (inset), (**b**) capacitance retention at different bending angles with the associated CV curves (inset), (**c**) cycling durability and coulombic efficiency over repeated GCD cycles, and (**d**) Ragone plot comparing the device with previously reported PPy/rGO-based ASCs. Reproduced with permission from [107]. Moyseowicz et al. prepared porous PPy/rGO hybrid electrodes using a hydrothermal self-assembly strategy, producing a highly porous polymer/graphene framework with an SSA of 597 m^2^ g^−1^ [108]. The optimized PPy/rGO hybrid delivered a specific capacitance of 198 F g^−1^ at 20 A g^−1^ and retained approximately 92% of its original capacitance after 3000 cycles. Furthermore, a hybrid capacitor employing a redox-active hydroquinone electrolyte achieved a specific energy of 6.5 Wh kg^−1^, highlighting the importance of porous architecture in enhancing ion diffusion and long-term electrochemical durability.

Nabeel et al. prepared a PPy/rGO composite electrode via a chemical oxidative polymerization method, in which rGO was incorporated during pyrrole polymerization to obtain a uniformly dispersed conductive network [106]. The optimized PPy/rGO electrode exhibited a specific capacitance of 375 F g^−1^ at 2 A g^−1^ (365. 1 F g^−1^ from CV at 10 mV s^−1^), together with a specific energy of 46.88 Wh kg^−1^ and a specific power of 1140.2 W kg^−1^, demonstrating an outstanding synergistic integration between PPy and rGO for enhanced charge storage. Similarly, Pawar et al. fabricated PPy/rGO composite thin film electrodes by a successive ionic layer adsorption and reaction (SILAR) technique, as illustrated in Figure 8i [107]. In brief, first rGO was deposited onto a stainless-steel substrate from aqueous dispersions of different concentrations, followed by the successive deposition of PPy through repeated SILAR cycles to achieve the desired film thickness. During film growth, pyrrole monomers were uniformly adsorbed onto the negatively charged rGO surface through electrostatic interactions, π–π stacking, and hydrogen bonding, while the oxygen-containing functional groups on rGO promoted strong interfacial adhesion and homogeneous PPy coating. The fabrication process of the flexible solid-state supercapacitor (FSSC) based on the rGO/PPy/PVA-H_2_SO_4_/WO_3_ configuration is schematically illustrated in Figure 8ii. The device consists of an rGO/PPy composite cathode, a WO_3_ anode, and a PVA-H_2_SO_4_ gel electrolyte assembled on a stainless-steel substrate to form an integrated flexible energy storage device. The layered architecture allowed PPy nanoparticles to be uniformly sandwiched between rGO sheets, resulting in a specific capacitance of 803 F g^−1^, which was substantially higher than pristine PPy (331 F g^−1^). The electrochemical performance and mechanical flexibility of the FSSC device are summarized in Figure 8iii. Electrochemical impedance spectroscopy (EIS) analysis (Figure 8iii(a)) showed relatively low impedance characteristics for the PPy/rGO electrode, consistent with improved electrical transport and favorable electrode/electrolyte interfacial behavior. However, quantitative comparison of charge-transfer resistance should be based on the fitted EIS parameters reported by the original study. The device also demonstrates excellent flexibility, retaining approximately 91% of its initial capacitance even at a bending angle of 160° with minimal distortion of the CV curves (Figure 8iii(b)). Long-term cycling tests show 79% capacitance retention and 90% coulombic efficiency after 5000 charge–discharge cycles (Figure 8iii(c)), confirming good electrochemical durability. Furthermore, the Ragone plot (Figure 8iii(d)) delivers a maximum energy density of 12 Wh kg^−1^ at a power density of 881 W kg^−1^, outperforming several previously reported PPy/graphene-based flexible supercapacitors.

Although binary PPy/rGO composites exhibit excellent conductivity and pseudocapacitive behavior, their electrochemical behavior can be further enriched through the inclusion of transition-metal oxides. Metal oxides provide abundant Faradaic redox-active moieties, increasing electrochemically available surface area, improving structural stability, and suppressing the mechanical degradation of PPy during prolonged cycling. The synergistic integration between PPy, rGO, and metal oxides significantly enhances specific capacitance, specific energy, rate capability, and cyclic durability. Copper oxide (CuO) has attracted significant interest because of its multiple oxidation states, high theoretical capacitance, low cost, eco-friendliness, and excellent redox activity. Incorporating CuO into PPy/rGO composites introduces additional Faradaic reactions, while rGO accelerates electron transport and PPy provides a flexible conducting matrix. Kandulna et al. synthesized PPy/CuO/rGO nanocomposites through pyrrole polymerization in the presence of CuO nanoparticles and rGO [109]. The optimized ternary composite exhibited a specific capacitance of 850.12 F g^−1^ at a sweep rate of 10 mV s^−1^. Although the original study also reported energy and power density values, these values are not included in the device-level quantitative comparison because the corresponding two-electrode device configuration and normalization basis have not been established.

Spinel ferrites such as NiCoFe_2_O_4_ may contribute additional Faradaic charge-storage processes through their accessible redox-active species. When incorporated into PPy/rGO composites, the ferrite phase and the PPy/rGO framework may influence electronic connectivity, interfacial properties, and structural stability. These proposed contributions should be interpreted in relation to the electrochemical and physicochemical evidence reported in the original study. Nabeel et al. synthesized a series of PPy/rGO/NiCoFe_2_O_4_ (PRNC) ternary composites through an auto-combustion method followed by chemical oxidative polymerization [110]. By optimizing the Ni/Co ratio, the PRNC80 electrode delivered the top electrochemical performance with a specific capacitance of 742.85 F g^−1^ (CV) and 585 F g^−1^ (GCD), significantly outperforming the other compositions, which was attributed by the original authors to the combined contributions of PPy, rGO, and NiCoFe_2_O_4_ nanoparticles.

MXenes have gained substantial attention as advanced two-dimensional (2D) electrode materials for next-generation SCs, owing to their high electrical conductivity, hydrophilic surface characteristics, abundant terminal functional groups, tunable interlayer spacing, and rich redox-active sites [111,112,113]. However, MXene nanosheets readily undergo self-restacking because of strong van der Waals interactions, resulting in reduced ion accessibility and inferior electrochemical behavior. The incorporation of PPy effectively expands the MXene interlayer spacing and introduces additional pseudocapacitance, while rGO constructs a three-dimensional conductive network that surpasses nanosheet agglomeration, enhances electrolyte penetration, and accelerates electron transport. Recently, Meng et al. synthesized a Ti_3_C_2_T_x_@PPy-rGO heterostructure hydrogel through in situ polymerization followed by low-temperature hydrothermal self-assembly [114].

**Figure 9:**
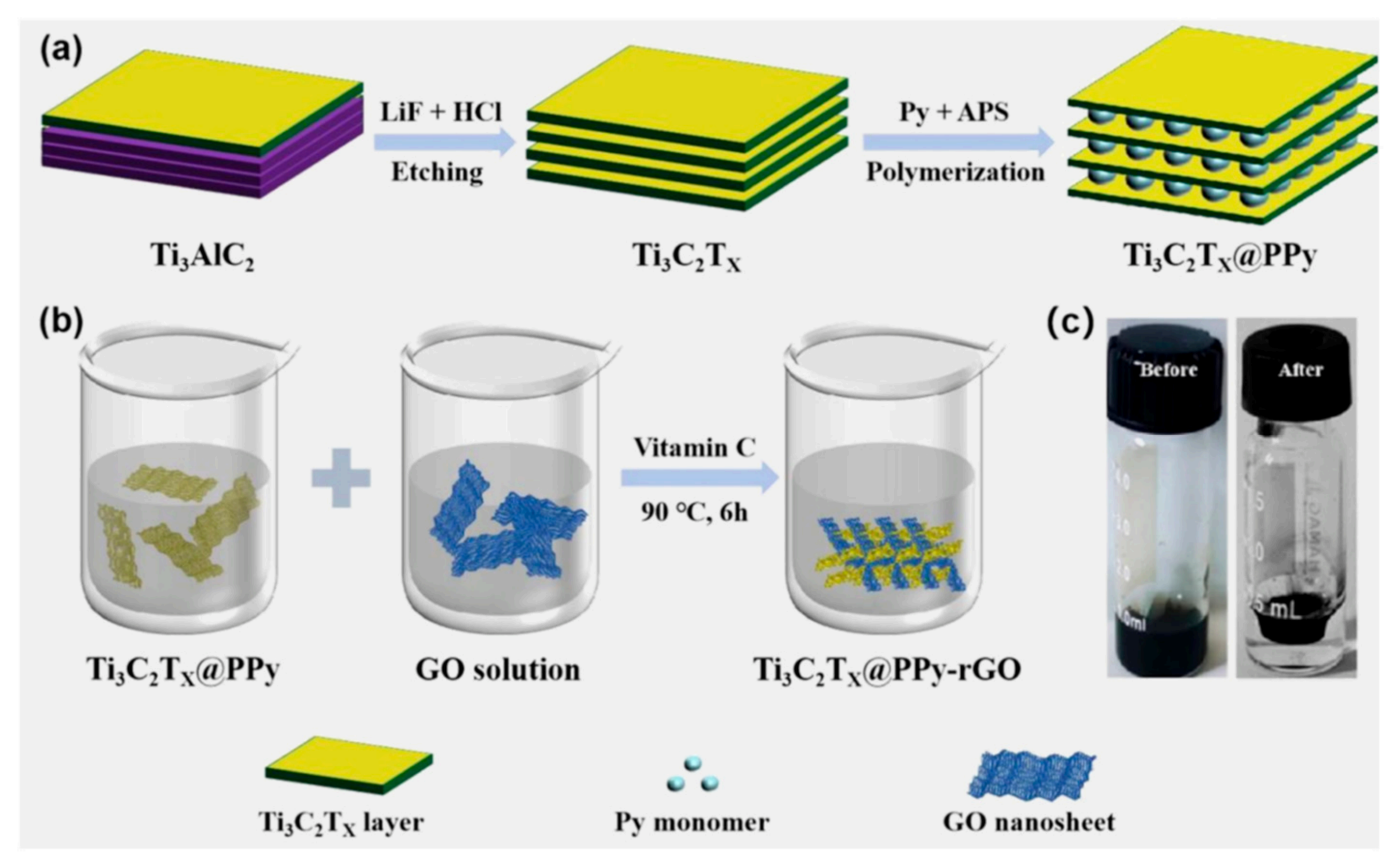
Schematic illustration of the synthesis of (**a**) Ti_3_C_2_T_x_@PPy through in situ OPA of pyrrole on MXene, (**b**) fabrication of the 3D Ti_3_C_2_T_x_@PPy-rGO hydrogel via hydrothermal self-assembly with GO and vitamin C reduction, and (**c**) photographs of the precursor suspension before and the hydrogel after gelation. Reproduced with permission from [114].

The construction method of the Ti_3_C_2_T_x_@PPy heterostructure and the successive Ti_3_C_2_T_x_@PPy-rGO hydrogel is illustrated in Figure 9. Initially, Ti_3_C_2_T_x_ MXene was acquired by selective etching of the MAX phase, followed by the in situ OPA of pyrrole using APS, resulting in the formation of Ti_3_C_2_T_x_@PPy with PPy uniformly anchored on the MXene layers (Figure 9a). Subsequently, the Ti_3_C_2_T_x_@PPy hybrid was dispersed in a GO suspension and subjected to mild hydrothermal treatment in the presence of vitamin C, which simultaneously reduced GO and promoted self-assembly into a three-dimensional porous Ti_3_C_2_T_x_@PPy-rGO hydrogel (Figure 9b). Vitamin C served as a safe reducing agent while minimizing MXene oxidation during hydrothermal processing. The resulting black hydrogel (Figure 9c) demonstrates the successful integration of PPy, MXene, and rGO into a stable, interconnected conductive network.

**Figure 10:**
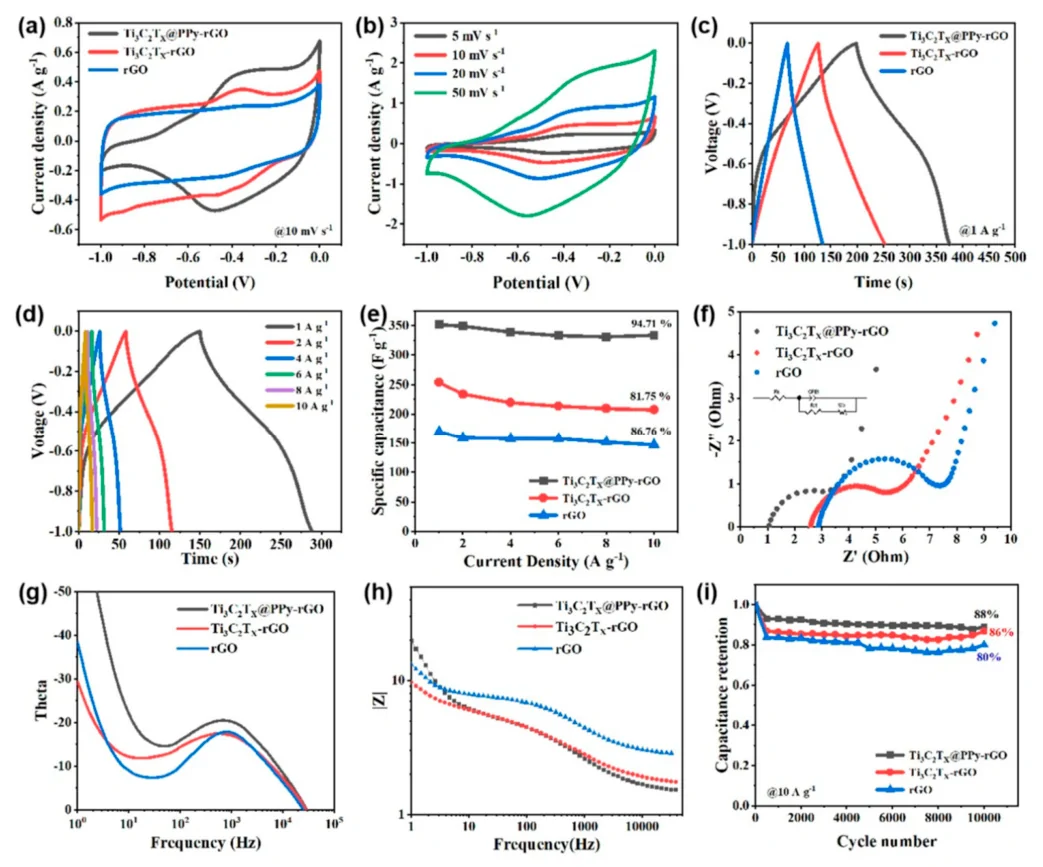
Electrochemical performance of rGO, Ti_3_C_2_T_x_-rGO, and Ti_3_C_2_T_x_@PPy-rGO: (**a**,**b**) CV, (**c**,**d**) GCD, (**e**) rate capability, (**f**) Nyquist plots, (**g**,**h**) Bode plots (phase angle and impedance magnitude), and (**i**) cycling durability over 10,000 GCD cycles. Reproduced with permission from [114].

The electrochemical behavior of rGO, Ti_3_C_2_T_x_-rGO, and Ti_3_C_2_T_x_@PPy-rGO was evaluated using cyclic voltammetry (CV) plots, galvanostatic charge and discharge (GCD) profiles, EIS results, and cycling stability, as summarized in Figure 10a–i. The Ti_3_C_2_T_x_@PPy-rGO electrode exhibits a major CV enclosed area and definite redox peaks (Figure 10a,b), demonstrating the integral contribution of EDLC and PSC. GCD measurements (Figure 10c,d) reveal longer discharge times and a high specific capacitance of 352.6 F g^−1^ at 1 A g^−1^, outperforming Ti_3_C_2_T_x_-rGO (254.2 F g^−1^) and rGO (170.3 F g^−1^). The composite also delivers excellent rate capability, retaining approximately 95% of its capacitance at 10 A g^−1^ (Figure 10e). EIS analysis (Figure 10f–h) showed reduced impedance characteristics, which are consistent with improved electrical transport and interfacial charge-transfer behavior. The enhanced electrochemical performance may also be associated with the interconnected porous architecture; however, quantitative attribution of the improvement to charge-transfer kinetics requires comparison of fitted resistance and diffusion-related parameters. Furthermore, the Ti_3_C_2_T_x_@PPy-rGO hydrogel exhibits excellent cycling durability, maintaining 88% of its original capacitance over 10,000 GCD cycles (Figure 10i). Superior electrochemical behavior is attributed to the synergistic effects of MXene, pseudocapacitive PPY, and the porous rGO network, which together accelerate quick charge transport, effective electrolyte diffusion, and abundant electroactive sites.

### 4.6. PEDOT/rGO Composites

PEDOT is one of the most attractive CPs for high-performance SCs owing to its excellent electrical conductivity, fast charge-transfer kinetics, high environmental stability, wide electrochemical potential window, and superior cycling durability compared with other CPs. Nevertheless, pristine PEDOT suffers from limited electrochemically active surface area and gradual structural degradation during repeated charge and discharge cycling, which restricts its achievable capacitance. rGO has emerged as an effective supporting material due to its high electrical conductivity, large SSA, outstanding mechanical robustness, and interconnected 2D conductive framework. The incorporation of rGO may help reduce graphene restacking and PEDOT aggregation, potentially improving electronic connectivity and electrolyte accessibility within the composite electrode. Subsequently, PEDOT/rGO composites exhibit significantly improved specific capacitance, rate capability, and cyclic durability compared with pristine PEDOT electrodes.

Ljubek et al. synthesized PEDOT/rGO composite electrodes through electrochemical polymerization followed by the electrochemical reduction of GO to rGO [115]. The successful reduction of GO significantly enhanced the electrochemical performance of the composite electrode, producing a specific capacitance of 74.67 F g^−1^ and a specific energy of 5.60 Wh kg^−1^. The SSC maintained 72–95% capacitance retention over 1000 charge–discharge cycles, demonstrating that electrochemically rGO effectively improves the conductivity and electrochemical activity of PEDOT-based electrodes. Abou Zeid et al. developed PEDOT/rGO nanohybrids through a green gamma-radiolysis synthesis performed under ambient conditions without catalysts or chemical oxidants [116]. The radiolytic process simultaneously reduced rGO and polymerized EDOT, yielding uniformly dispersed PEDOT on rGO sheets with improved structural ordering and conductivity. The optimized PEDOT/rGO nanohybrid achieved a specific capacitance of approximately 248 F g^−1^, which was substantially higher than many conventional PEDOT-based electrodes, a result the original authors attributed to the combined contributions of the conductive rGO network and the pseudocapacitive PEDOT matrix.

Although PEDOT/rGO binary composites exhibit improved conductivity and cycling stability, their electrochemical performance can be further enhanced by incorporating transition-metal oxides. Metal oxides may contribute additional Faradaic charge-storage processes, while rGO may provide electronic connectivity within the composite and PEDOT may provide a flexible polymer matrix that influences the structural response of the composite during cycling. Consequently, ternary PEDOT/rGO/metal oxide composites generally deliver superior capacitance, enhanced rate capability, and higher cycling durability. Chromium oxide (Cr_2_O_3_) possesses high theoretical capacitance and excellent electrochemical activity, whereas zirconia contributes structural stability and improved durability. The combination of Cr_2_O_3_-ZrO_2_ nanoparticles with PEDOT/rGO creates a robust hybrid electrode capable of efficient electron and ion transport. Benchikh et al. prepared a PEDOT:PSS/Cr_2_O_3_-ZrO_2_-rGO quaternary composite using co-precipitation followed by chemical oxidative polymerization [117]. The constructed electrode exhibited a specific capacitance of 720.25 F g^−1^ at 1 A g^−1^, together with a specific energy of 39.7 Wh kg^−1^ and a specific power of 365.6 W kg^−1^. Moreover, the electrode retained 90.9% of its capacitance over 8000 cycles, while the assembled asymmetric device delivered 282.7 F g^−1^ with 85.7% capacitance preservation after 8000 cycles, with the observed performance attributed by the original authors to the synergistic effect among PEDOT, rGO, and mixed metal oxides.

Copper (I) oxide (Cu_2_O) is an attractive pseudocapacitive material because of its versatile valence states, cost-effectiveness, and rich redox chemistry. Incorporation of Cu_2_O into PEDOT/rGO composites introduces additional Faradaic charge-storage reactions, while the graphene network accelerates electron transport. Kanathedath et al. fabricated Cu_2_O/graphene/PEDOT:PSS 3D flexible electrodes using a polyurethane foam-supported architecture for structural energy storage applications [118].

**Figure 11:**
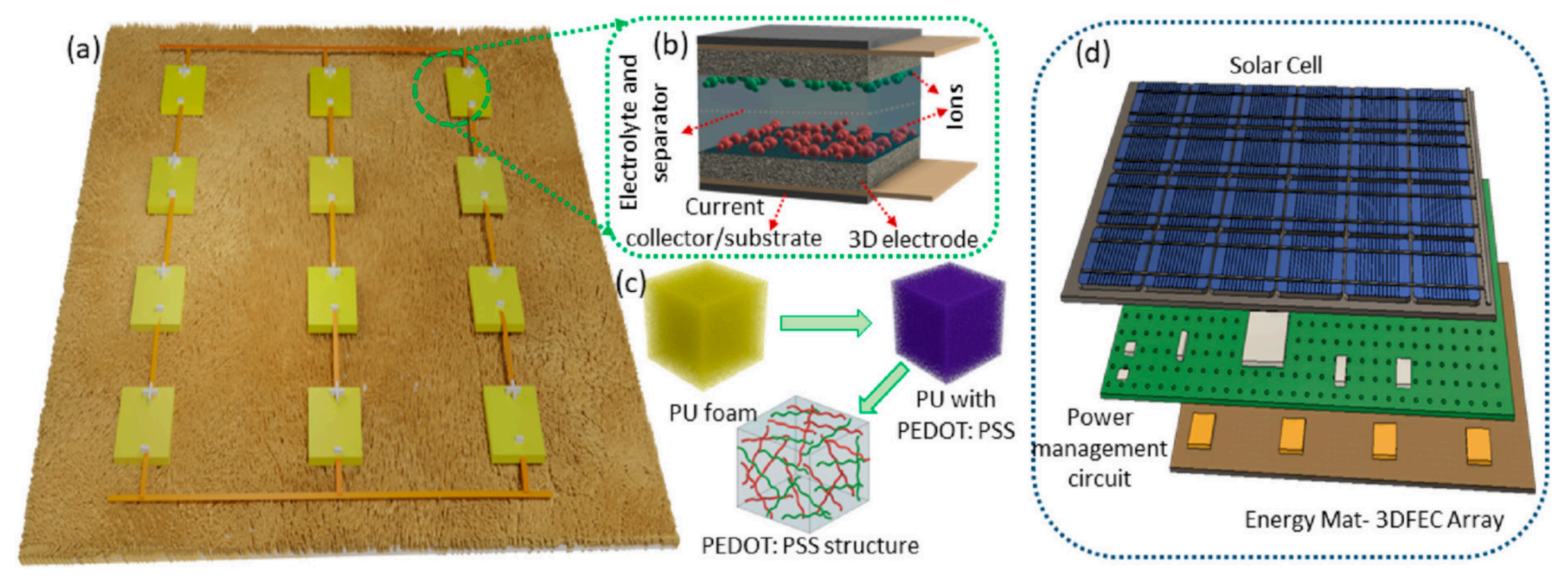
Pictorial design of (**a**) the flexible 3D-FEC energy mat, (**b**) the layered architecture of an individual 3D-FEC, (**c**) preparation of PEDOT:PSS-coated polyurethane foam, and (**d**) integration of the energy mat with a solar cell and power management circuit for sustainable energy storage. Reproduced with permission from [118].

The fabrication and application of the 3D flexible energy capacitor (3D-FEC) are illustrated in Figure 11. Multiple 3D-FEC units were combined into a cellulose-polyester textile to produce a flexible energy storage mat (Figure 11a), while the layered electrode configuration of an individual device is shown in Figure 11b. The mechanical stability of the energy mat was evaluated under applied static loads to examine its electrochemical performance. For practical operation, the energy mat was coupled with a solar cell and power management circuit, enabling efficient harvesting and storage of solar energy in the interconnected 3D-FEC array (Figure 11d).

**Figure 12:**
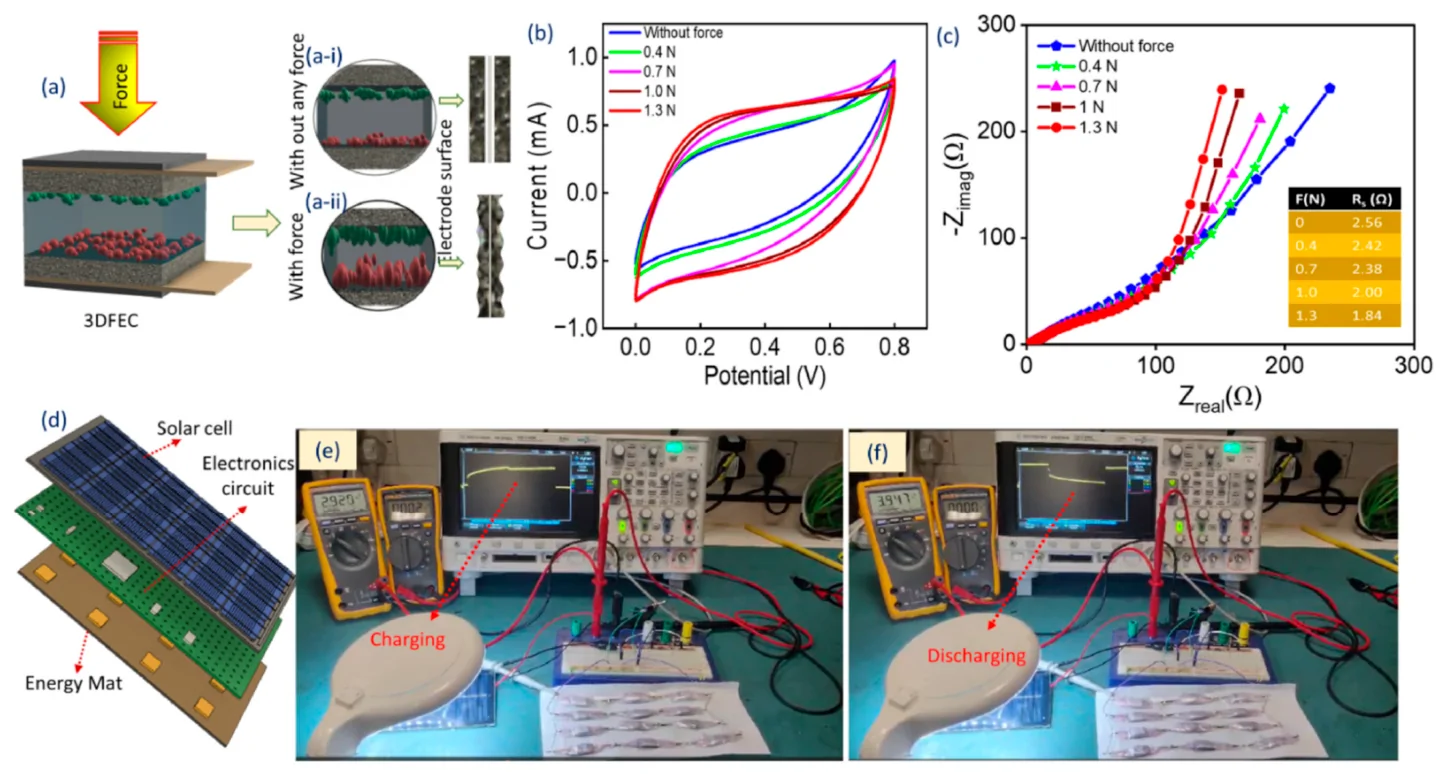
Pressure-dependent electrochemical performance and solar-powered operation of the 3D-FEC energy mat: (**a**) schematic of structural changes under applied force, (**b**) CV curves under different loading conditions, (**c**) Nyquist plots at various applied forces, (**d**) integration of the energy mat with a solar cell and power management circuit, and (**e**,**f**) photographs illustrating the charging and discharging processes. Reproduced with permission from [118].

The 3D-shaped flexible electrochemical capacitor exhibited a volumetric capacitance of 1.09 F cm^−3^ (1.17 F g^−1^) at 0.25 mA cm^−2^, with volumetric and gravimetric energy densities of 94.15 μWh cm^−3^ and 0.102 Wh kg^−1^, respectively. The corresponding power densities were 0.985 mW cm^−3^ and 1.06 W kg^−1^, respectively. The hierarchical porous structure also showed excellent mechanical strength, making it suitable for structural supercapacitors integrated with flexible electronic systems. The pressure-dependent electrochemical behavior and practical application of the 3D-FEC energy mat are presented in Figure 12. The device was evaluated under different external loads to examine the influence of mechanical deformation on its electrochemical performance (Figure 12a). CV and EIS analyses (Figure 12b,c) reveal that increasing the applied load enhances the capacitance while reducing the internal resistance, owing to improved electrode–electrolyte contact and shortened ion transport pathways. Finally, the energy mat was integrated with a solar cell and a power management circuit (Figure 12d–f), demonstrating efficient solar-powered charging and discharging, highlighting its potential for flexible structural energy storage applications.

Transition metal sulfides (TMSs) generally exhibit higher electrical conductivity and quicker redox kinetics than their oxide counterparts because sulfur possesses lower electronegativity than oxygen. Incorporating a metal sulfide into a PEDOT/rGO composite may introduce additional charge-storage contributions and modify the electronic connectivity and interfacial characteristics of the electrode. Hafnium disulfide (HfS_2_), a layered transition-metal dichalcogenide, possesses excellent electrical conductivity, ample exposed active sites, and swift ion-diffusion channels. When incorporated into PEDOT/rGO composites, HfS_2_ effectively enhances pseudocapacitance while rGO prevents nanosheet aggregation and PEDOT provides a flexible conductive framework.

Ganesan et al. synthesized PEDOT/rGO/HfS_2_ ternary nanocomposites through a combination of hydrothermal synthesis and in situ polymerization [119]. The composite electrode exhibited a reported specific capacitance of 1015 F g^−1^ at 5 A g^−1^. Moreover, the prepared electrode maintained 98.1% of its original capacitance over 10,000 GCD cycles, indicating its cycling stability under the reported experimental conditions.

Greco et al. developed an eco-friendly PEDOT:PSS/Edge oxidized graphite (EOG) ternary nanocomposite by incorporating sustainably synthesized EOG with green additives (glucose and ascorbic acid) to fabricate flexible supercapacitor electrodes [120]. Unlike conventional graphene oxide prepared through harsh chemical oxidation processes, EOG was synthesized using hydrogen peroxide, offering a potentially more environmentally benign alternative while retaining oxygen-containing functional groups at the graphite edges that facilitate strong interfacial interactions with the PEDOT:PSS matrix. The addition of EOG together with ascorbic acid significantly improved filler dispersion, surface homogeneity, thermal stability, and charge transport, resulting in reduced charge-transfer resistance and enhanced ion diffusion. The electrochemical performance of PEDOT nanocomposite electrodes was evaluated using both a conventional and three-electrode configuration and symmetric two-electrode cells. The specific capacitance was evaluated from CV measurements in the three-electrode configuration, with the AA-EOG/PEDOT:PSS composite exhibiting a 4.75% increase in specific capacitance compared with pristine PEDOT:PSS at a scan rate of 25 mV s^−1^. The authors also conducted GCD measurements using symmetric cells within a voltage window of 0 to 0.8 V. Based on these device measurements, the energy and power densities of 2.9 Wh kg^−1^, and a specific power of 0.24 W kg^−1^, respectively. However, the absolute specific capacitance of the symmetric device and the precise mass-normalization basis used for the energy and power calculations were not explicitly reported. The original authors attributed the improved electrochemical performance to interactions among PEDOT:PSS, EOG, and the additives. However, the individual contributions of these interactions to electrochemically active surface area and ion transport should be interpreted in relation to the supporting characterization evidence.

Hybridization of two intrinsically conducting polymers has appeared as an efficient technology for enhancing the electrochemical behavior of supercapacitor electrodes by combining the complementary advantages of each polymer. PEDOT provides excellent electrical conductivity, environmental stability, and rapid electron transport, whereas PPy contributes ample redox-active moieties and high pseudocapacitance. Combining PEDOT and PPy may influence charge transport, electrochemical accessibility, and structural behavior during cycling. However, the individual contributions of PEDOT-PPy interactions require appropriate experimental evidence and should not be inferred from capacitance improvement alone. Kocyiğit et al. fabricated PEDOT/PPy hybrid electrodes via electrochemical copolymerization in the presence of pyrimidine-N-glycoside dopants on pencil graphite electrodes (PGE) [121]. Among the prepared electrodes, the PEDOT/PPy-2/PGE electrode exhibited the best electrochemical behavior, delivering a specific capacitance of 195.2 F g^−1^ in a three-electrode set-up. Furthermore, the assembled SSC attained a specific capacitance of 61.8 F g^−1^ and a specific energy of 3.7 Wh kg^−1^, indicating that molecular doping together with PEDOT-PPy copolymerization effectively improves charge transport and electrochemical energy storage.

Recently, Shetty et al. extended this concept by developing a screen-printable water-based PEDOT:PSS/PPy/rGO conductive ink for flexible micro-supercapacitors [122]. In this ternary hybrid system, PEDOT:PSS served as the conductive polymer matrix, PPy supplied additional pseudocapacitive redox sites, whereas rGO constructed a highly conductive network that facilitated electron transport and prevented polymer aggregation. Hydroxyethyl cellulose (HEC) was employed as a binder material to improve the compatibility between hydrophobic PPy and hydrophilic PEDOT:PSS, resulting in a homogeneous, stable, and printable conductive ink. The prepared PEDOT:PSS/PPy/rGO electrode displayed an areal capacitance of 219.7 mF cm^−2^ at 5 mV s^−1^, which was significantly higher than the PEDOT:PSS/PPy binary electrode (193.16 mF cm^−2^), together with a specific energy of 8.45 μWh cm^−2^ and a specific power of 21.45 μW cm^−2^. The higher areal capacitance indicates improved charge-storage performance under the reported test conditions. The proposed contributions of the individual components to electrical conductivity, ion transport, and structural stability should nevertheless be interpreted in light of the characterization evidence available in the original study. Table 3 provides a comparison of the electrochemical performance of CP/rGO composite electrodes in terms of specific capacitance and related parameters.

**Table 3:** Comparative electrochemical performance of CP/rGO composite electrodes in terms of specific capacitance.

S. No.	Material	Electrolyte	Specific Capacitance/Specific Capacity (3-Electrode Configuration) and Mass Loading (mg/cm^2^)	Specific Capacitance (2-Electrode Configuration) and Mass Loading (mg)	Energy and Power Density	Capacitance Retention/Stability (No. of Cycles at Current Density or Scan Rate)/Potential Window	[Ref]
1	PANI/rGO	1 M H_2_SO_4_	420 F g^−1^ at 0.2 A g^−1^ (Mass: ---)	220 F g^−1^ at 0.2 A g^−1^ (SSC device)	9.3 Wh kg^−1^ and 0.1 kW kg^−1^	80% capacitance retention after 6000 GCD cycles at 2 A g^−1^/ΔV = 0.0–1.1 V	[89]
2	PANI/rGO	6 M KOH	704 F g^−1^ at 1 A g^−1^ (Mass: 5.3 mg cm^−2^)	58 F g^−1^ at 1 A g^−1^ (ASC device)	23.3 Wh kg^−1^ and 850 W kg^−1^	67% capacitance retention after 1000 GCD cycles at 5 A g^−1^/ΔV = 0.0–1.7 V	[90]
3	rGO/PANI/MnO_2_	1 M H_2_SO_4_	740 F g^−1^ at 1 A g^−1^ (Mass: ---)	----	----	97% capacitance retention after 5000 GCD cycles at 15 A g^−1^/ΔV = −0.2–0.8 V	[103]
4	rGO/TiO_2_/PANI	1 M H_2_SO_4_	252.52 F g^−1^ at 0.5 A g^−1^ (Mass: ---)	----	----	89.9% capacitance retention after 3000 GCD cycles at 0.5 A g^−1^/ΔV = −0.2–0.8 V	[104]
5	PANI/Cl-GO/PbO_2_	1 M H_2_SO_4_ and 1 M KOH		ASC 97 mF cm^−2^ at 10 mV s^−1^ (acidic electrolyte); 54 mF cm^−2^ at 10 mV s^−1^ (alkaline electrolyte)	----	----	[105]
6	PPy/rGO	3 M KOH	375 F g^−1^ at 2 A g^−1^ (Mass: 1 mg cm^−2^)	----	----	----	[106]
7	PPy/rGO	1 M H_2_SO_4_	803 F g^−1^ at 5 mV s^−1^	56 F g^−1^ at 5 mV s^−1^ (ASC device); Mass ratio 0.37	12 Wh kg^−1^ and 881 W kg^−1^	79% capacitance retention after 5000 GCD cycles at 1.2 A g^−1^/ΔV = 0.0–1.3 V	[107]
8	PPy/rGO-HT hybrid	1 M H_2_SO_4_	186 mAh g^−1^ at 0.2 A g^−1^	60 F g^−1^ at 0.2 mV s^−1^ (SSC device)	6.5 Wh kg^−1^ and 0.08 kW kg^−1^	104% capacitance retention after 12,000 GCD cycles at 0.2 A g^−1^/ΔV = 0.0–0.9 V	[108]
9	PPy/CuO/rGO	1 M H_2_SO_4_	773.68 F g^−1^ at 0. 7 A g^−1^		----	----	[109]
10	PPy/rGO/NiCoFe_2_O_4_	3 M KOH	585 F g^−1^ at 1 A g^−1^		----	----	[110]
11	Ti_3_C_2_T_x_/PPy/rGO	1 M KOH	352.57 F g^−1^ at 1 A g^−1^ (Mass: 1 mg cm^−2^)	----	----	88% capacitance retention after 10,000 GCD cycles at 10.0 A g^−1^/ΔV = −1.0–0.0 V	[114]
12	PEDOT/rGO	1 M KCl	----	74.67 F g^−1^ at 1.5 A g^−1^ (SSC device)	5.60 Wh kg^−1^ and 26.19 kW kg^−1^	72% capacitance retention after 1000 GCD cycles/ΔV = −0.8–0.8 V	[115]
13	PEDOT/rGO	1 M KOH	~248 F g^−1^ at 10 mV s^−1^	----	----	----	[116]
14	PEDOT:PSS/Cr_2_O_3_-ZrO_2_-rGO	6 M KOH	720.25 F g^−1^ at 1 A g^−1^	282.7 F g^−1^ (ASC device)	39.7 Wh kg^−1^ and 365.6 W kg^−1^	85.7% capacitance retention after 8000 GCD cycles at 1 A g^−1^	[117]
15	PEDOT:PSS/Cu_2_O-Graphene	3 M NaCl	----	1.09 F cm^−3^ at 0.25 mA cm^−2^	94.15 μWh cm^−3^ and 0.985 mW cm^−3^	----	[118]
16	PEDOT/rGO-HfS_2_	3 M KOH	1015 F g^−1^ at 5 A g^−1^	----	----	98.1% capacitance retention after 10,000 GCD cycles at 5 A g^−1^/ΔV = 0.2–1.2 V	[119]
17	PEDOT:PSS/EOG-AA	0.01 M PBS	----	----	2.9 Wh kg^−1^ and 0.24 W kg^−1^	94.6% capacitance retention after 1000 GCD cycles	[120]
18	PEDOT/PPy-PGE	0.5 M PVA/H_2_SO_4_ gel	195.2 F g^−1^ at 250 μA	61.8 F g^−1^ at 250 μA (SSC device)	3.7 Wh kg^−1^ and ----	----	[121]
19	PEDOT:PSS/PPy-rGO	----		219.7 mF cm^−2^ at 5 mV s^−1^	8.45 μWh cm^−2^ and 21.45 μW cm^−2^	96.1% capacitance retention after 10,000 GCD cycles	[122]

Note: ---- indicates not available or not reported in the original publications.

## 5. Challenges and Research Gaps

Although remarkable advances have been achieved in polymer/graphene-based composites for supercapacitor electrodes, several unresolved scientific and engineering issues hinder their transition from laboratory-scale research to commercial energy storage systems. Among these, the limited cycling durability of conducting polymers remains one of the most significant concerns. Repeated ion insertion/extraction throughout successive charge–discharge cycles induces continuous volumetric expansion and contraction of polymer chains, resulting in structural distortion, crack formation, mechanical degradation, and progressive loss of electrochemical activity. The incorporation of graphene has been shown to improve structural integrity and electrical conductivity; however, maintaining stable capacitance over prolonged cycling remains a considerable challenge.

Another critical limitation arises from the intrinsic tendency of graphene nanosheets to undergo aggregation and layer restacking because of strong π–π interactions and van der Waals forces. Such restacking substantially decreases the effective surface area available for charge storage while restricting electrolyte penetration and ion diffusion pathways. Consequently, the design of efficient approaches that suppress graphene aggregation without compromising its outstanding electrical conductivity remains a key research priority.

The performance of polymer/graphene composites is also strongly influenced by the nature of the interface between the two constituents. Mechanistic interpretations of the electrochemical enhancement should, however, be distinguished from directly measured evidence. An increase in specific capacitance, improved rate capability, or a favorable electrode morphology alone does not conclusively demonstrate faster charge-transfer kinetics, enhanced ion diffusion, or a larger electrochemically active surface area. Such interpretations should preferably be supported by quantitative electrochemical and physicochemical measurements, including EIS, charge-transfer resistance (R_ct_), equivalent series resistance (ESR), diffusion-rate parameters, electrochemically active surface area, or other independent characterization techniques. Similarly, a small circle in a Nyquist plot may indicate lower impedance or charge-transfer resistance, but it should not by itself be regarded as complete evidence of enhanced reaction kinetics without appropriate circuit fitting and quantitative comparison. Therefore, throughout this review, mechanistic explanations are presented as interpretations consistent with the reported experimental evidence when quantitative supporting data is unavailable, rather than as definitive causal conclusions. Nevertheless, the mechanisms governing electron transport, ion migration, interfacial adhesion, and synergistic electrochemical interactions have not yet been fully clarified. Comprehensive investigations using advanced in situ characterization techniques together with computational modeling are therefore essential to establish a deeper understanding of the structure–property–performance relationships in these hybrid materials.

Large-scale production presents another important obstacle to practical implementation. Numerous synthesis methods reported in the literature rely on sophisticated processing techniques, expensive raw materials, or laboratory-scale procedures that are difficult to adapt for industrial manufacturing. In addition, variations in electrode fabrication methods, electrolyte composition, mass loading, and electrochemical evaluation protocols often hinder meaningful comparisons among different studies. Therefore, the adoption of standardized fabrication procedures and universally accepted performance evaluation criteria is crucial for accurately assessing newly developed electrode materials.

Furthermore, while many investigations primarily emphasize high specific capacitance, relatively fewer studies evaluate practical device characteristics such as energy density, power capability, mechanical durability, operational safety, and long-term reliability under realistic working conditions. Future research should therefore prioritize the development and evaluation of full-cell supercapacitor devices using practical electrode loadings and extended cycling tests rather than relying exclusively on three-electrode measurements.

Practical electrode loading and device-level normalization are critical considerations when assessing the real-world performance of SC electrodes. High gravimetric capacitance measured at low active material loading does not necessarily translate into high areal, volumetric, or packaged-device performance. Practical evaluation should therefore report the active material loading (mg cm^−2^), electrode thickness, electrode density, active-to-inactive mass ratio, and the mass contributions of current collectors, separators, electrolyte, and packaging. For asymmetric and hybrid supercapacitors, appropriate charge and mass balancing between the positive and negative electrodes is also essential to maximize the usable voltage window while avoiding polarization and side reactions. Device-level energy and power densities should clearly state the mass basis used for normalization and indicate whether current collectors, separators, electrolyte, and packaging are included. Accordingly, three-electrode measurements, particularly those obtained at low active-material loading, should primarily be regarded as materials-screening results rather than direct evidence of commercial device performance. Demonstration using realistic mass loading, two-electrode full cell configurations, and packaged-cell normalization is therefore necessary to substantiate commercialization claims.

### Technical Challenges and Requirements for Industrial Implementation

(i) Although polymer/graphene composites have demonstrated promising electrochemical performance in laboratory-scale studies, several technical and engineering challenges must be addressed before their widespread implementation in commercial supercapacitor devices. A major limitation is the scalability and reproducibility of electrode material synthesis. Many reported preparation methods involve multistep processing, hydrothermal treatment, chemical oxidation, high-temperature annealing, or the use of expensive and hazardous reagents. Although these approaches can produce well-defined nanostructures at the laboratory scale, their large-scale implementation may be limited by high energy consumption, batch-to-batch variation, complicated process control, and difficulties in maintaining uniform material properties. Therefore, scalable synthesis strategies should provide consistent composition, morphology, defect density, conductivity, and electrochemical performance while reducing processing time, energy requirements, and production costs.
(ii) The reproducible formation of the composite electrode is another important challenge. The electrochemical properties of polymer/graphene electrodes are strongly influenced by the active material loading, thickness, distribution of the composite, binder content, solvent selection, drying conditions, and interaction with the current collector. In laboratory studies, relatively low active material loadings are frequently used to obtain high gravimetric capacitance values. However, these conditions may not accurately represent practical electrodes, which generally require higher active material loading while maintaining sufficient electronic conductivity, electrolyte penetration, mechanical integrity, and ion transport. Increasing electrode thickness can also generate internal diffusion limitations and higher electrical resistance. Consequently, future studies should report the active material loading in mg cm^−2^, electrode thickness, electrode density, and the mass contribution of inactive components.
(iii) The selection of binders and conductive additives also requires careful consideration. Polymeric binders such as PVDF can improve electrode adhesion and mechanical integrity, but excessive binder content may block active sites, reduce electrolyte accessibility, and increase the internal resistance of the electrode. In addition, the use of organic solvents such as NMP can increase manufacturing costs and raise environmental and occupational safety concerns. Binder-free electrodes, water-processable binders, and environmentally preferable solvents may provide more sustainable alternatives. Nevertheless, these alternatives must be evaluated in terms of adhesion strength, long-term stability, compatibility with the active material, and large-scale processing capability.
(iv) The contribution of current collectors and other inactive components should also be carefully considered when evaluating the practical performance of polymer/graphene composite electrodes. Conductive substrates such as nickel foam, stainless steel, titanium, and carbon-based current collectors can improve electron transport and facilitate the deposition of active materials. However, the mass, porosity, surface roughness, and electrochemical activity of the current collector may influence the measured capacitance. Therefore, the reported electrochemical values should clearly indicate whether the capacitance is calculated using the mass of the active material alone, the complete electrode, or the total device. Such reporting is essential for establishing meaningful comparisons between different studies.
(v) Another critical issue is the long-term structural and electrochemical stability of CPs. CPs such as PANI, PPy, and PEDOT can undergo repeated swelling, contraction, doping, and de-doping during charge–discharge operation. These processes may cause mechanical cracking, loss of electrical contact, polymer detachment, and gradual deterioration of the electrode structure. Graphene can improve the mechanical integrity and electrical conductivity of the composite; however, the extent of this improvement depends on the interfacial integration, graphene distribution, polymer morphology, and composite architecture. The long-term stability of the polymer/graphene interface should therefore be investigated using extended cycling tests, post-cycling morphological analysis, and electrochemical impedance measurements.
(vi) Electrolyte selection influences the operating voltage, energy density, cost, safety, and durability of SCs. Aqueous electrolytes are economical and conductive but have a limited voltage window, whereas organic and ionic liquid electrolytes offer wider voltage ranges but may involve higher cost, toxicity, flammability, viscosity, and environmental concerns. Electrolyte compatibility, leakage, gas generation, drying, and long-term conductivity must be carefully evaluated.
(vii) Asymmetric and hybrid SCs require appropriate balancing of positive and negative electrodes to prevent polarization, side reactions, and degradation. Practical device design should consider electrode mass, charge-storage capacity, electrolyte stability, and operating voltage. Therefore, full cell testing with realistic mass loading, energy and power density, leakage current, self-discharge, and long-term cycling is essential.
(viii) Industrial development requires reliable packaging and consistent manufacturing processes that can withstand temperature, humidity, mechanical stress, and repeated operation. Scalable techniques such as coating, drying, calendaring, stacking, and winding should be considered. Quality control should monitor electrode thickness, material distribution, adhesion, internal resistance, leakage current, capacitance, and cycle life.
(ix) Future studies should focus on practical electrode loading, scalable manufacturing, device-level performance, environmental safety, cost analysis, and long-term reliability rather than only reporting high specific capacitance under laboratory conditions.

## 6. Future Perspectives and Conclusions

Future developments in polymer/graphene-based supercapacitor electrodes are expected to emphasize the rational engineering of hierarchical, multifunctional architectures capable of simultaneously achieving high energy density, superior power output, rapid charge transport, and outstanding cycling durability. The integration of additional electroactive components, including transition metal oxides, MXenes, metal–organic frameworks (MOFs), and carbon nanotubes, may offer opportunities to modify the electrochemical properties of polymer/graphene composites. Depending on the material composition and architecture, these components may contribute to charge-storage activity, electronic connectivity, and electrolyte-ion accessibility. Systematic comparisons and appropriate quantitative characterization should establish their individual contributions and any synergistic effects.

Considerable attention is also likely to be directed toward the fabrication of flexible, wearable, and deformable energy storage devices to satisfy the growing demand for next-generation portable and wearable electronics. Emerging manufacturing technologies, such as electrospinning, additive manufacturing, three-dimensional printing, and other advanced fabrication technologies, offer promising opportunities for producing lightweight, mechanically resilient, and highly efficient supercapacitor electrodes with complex architectures.

In parallel, artificial intelligence (AI), machine learning (ML), and computational materials modeling are expected to transform the discovery and optimization of advanced electrode materials. Data-driven methodologies can efficiently screen candidate materials, predict electrochemical behavior, optimize structural parameters, and identify suitable processing conditions, thereby significantly reducing experimental time, cost, and resource consumption.

Sustainability will remain an increasingly important consideration in future research. Greater emphasis should be placed on environmentally friendly synthesis strategies, renewable polymer precursors, and green manufacturing processes to minimize environmental impact. The utilization of biomass-derived polymers together with sustainable graphene production technologies offers an attractive pathway toward environmentally responsible and economically viable energy storage systems.

Overall, polymer/graphene composites have demonstrated exceptional potential as next-generation electrode materials for high-performance supercapacitors because of the complementary characteristics and strong synergistic interactions between conducting polymers and graphene. Significant progress has been achieved in composite design, synthesis methodologies, structural optimization, and electrochemical performance over the past decade. Nevertheless, several challenges, including limited long-term stability, scalable manufacturing, cost-effective production, and practical device integration, must be addressed before these materials can achieve widespread commercial adoption. Continued interdisciplinary collaboration among researchers in materials science, electrochemistry, nanotechnology, computational modeling, and engineering will play a crucial role in overcoming these limitations and accelerating the development of advanced supercapacitor technologies capable of meeting future global energy storage requirements.

## Figures and Tables

**Table 1:** Comparison of EDLCs, PCs, and HSCs.

Types of SCs	Typical Electrode Materials	Charge Storage Mechanism	Advantages	Limitations	Typical Applications
Electrical double-layer capacitors (EDLCs)	Carbonaceous materials such as AC, graphene, CNTs, CNFs, porous carbon, carbon aerogels, carbon cloth, and biomass-derived carbons	Charge accumulation through the formation of an electrochemical double layer (non-Faradaic process)	Outstanding specific power, exceptional rate capability, long-term durability, superior reversibility, high coulombic efficiency, excellent chemical and thermal stability, low maintenance requirements	Limited energy density because energy storage is restricted to surface charge accumulation. The specific capacitance is generally lower than that of pseudocapacitive materials. Performance strongly depends on pore architecture and electrolyte ion diffusion	Regenerative braking systems, backup power supplies, memory protection, pulse power delivery, load leveling, renewable energy storage, and portable electronic devices
Pseudocapacitors (PCs)	TMOs (RuO_2_, MnO_2_, NiO, Co_3_O_4_, Fe_2_O_3_, V_2_O_5_), transition metal sulfides, phosphides, nitrides, MXenes, and intrinsically conducting polymers such as PANI, PPy, and PEDOT	Charge storage through Faradaic redox reactions (Faradaic process)	Supreme specific capacitance, high specific energy, fast redox kinetics, abundant electrochemically active sites, enhanced electrochemical performance than EDLCs	Lower cycling stability due to repeated structural elongation and shrinkage during redox reactions, relatively poor electrical conductivity for many metal oxides, limited rate capability in some materials, and possible dissolution or degradation of conducting polymers during prolonged cycling	High-performance portable electronics, wearable devices, electric vehicles, hybrid energy storage systems, and high energy electrochemical storage devices
Hybrid supercapacitors (HSCs)	Asymmetric combinations of carbon materials with pseudocapacitive materials, including activated carbon//metal oxides, graphene//conducting polymer, CNT//metal sulfides, carbon//MXene, biomass-derived porous carbon//transition metal compounds, and other composite electrodes	Charge storage via Faradaic and non-Faradaic processes	Balanced electrochemical performance with higher specific energy than EDLCs and better specific power and cycling stability than PCs. Wide operating voltage, improved capacitance, enhanced rate capability, and better utilization of active materials	More complicated electrode design and fabrication, possible imbalance between positive and negative electrodes, optimization of mass loading and charge balance is required, and long-term stability depends on electrode compatibility and electrolyte selection	Electric vehicles. Hybrid electric systems, renewable energy storage, smart grids, aerospace electronics, medical devices, consumer electronics, and large-scale power management systems

**Table 2:** Common electrochemical equations used to evaluate supercapacitor performance.

Parameter	Equation	Unit
**Specific** **capacitance (3-electrode)**	$C_{s}=\frac{I\Delta t}{m\Delta V}$ (1)	F g^−1^
**Areal capacitance**	$C_{A}=\frac{I\Delta t}{A\Delta V}$ (2)	F cm^−2^
**Volumetric capacitance**	$C_{V}=\frac{I\Delta t}{V\Delta V}$ (3)	F cm^−3^
**Specific capacity**	$Q_{s}=\frac{I\Delta t}{3.6m}$ (4)	mAh g^−1^
**Specific capacity**	$Q_{s}=\frac{I\Delta t}{m}$ (5)	C g^−1^
**Specific capacitance from CV**	$C=\frac{\oint |I|\mathrm{dV}}{2\mathrm{mv}\Delta V}$ (6)	F g^−1^
**Device capacitance (2-electrode)**	$C_{\mathrm{cell}}=\frac{I\Delta t}{m_{T}\Delta V}$ (7)	F g^−1^
**Energy density (2-electrode)**	$E=\frac{1}{2}\frac{C{\left(\Delta V\right)}^{2}}{3.6}$ (8)	Wh kg^−1^
**Power density (2-electrode)**	$P=\frac{3600E}{\Delta t}$ (9)	W kg^−1^
**Coulombic efficiency**	$\eta =\frac{t_{d}}{t_{c}}\;\times \;100$ (10)	%
**Mass balance (2-electrode; Asymmetric device)**	${\frac{m}{m^{-}}}^{+}=\frac{C^{-}{\Delta V}^{-}}{C^{+}{\Delta V}^{+}}$ (11)	---

Symbols: C_s_ = specific capacitance of a single electrode (F g^−1^); C_cell_ = capacitance of the full device (F g^−1^); I = applied current (A); Δt = discharge time (s); m = active mass of the single electrode (g); m_T_ = total active mass of both electrodes (g); ΔV = operating potential or voltage window, excluding IR drop (V); ∮∣*I*∣*dV* = integral of the absolute current over the complete anodic–cathodic CV loop; the factor 2 corresponds to full-loop integration; v = scan rate (mV s^−1^); m^+^, m^−^, = active masses of the positive and negative electrodes (g); C^+^, C^−^ and ΔV^+^, ΔV^−^ = their respective specific capacitances and potential windows; E = energy density (Wh kg^−1^; divide by 3.6 to convert from J g^−1^); P = power density (W kg^−1^); η = coulombic efficiency; t_d_, t_c_ = discharge and charge times (s). For battery-type electrodes, specific capacity (C g^−1^ or mAh g^−1^) should be used in place of capacitance.

## Data Availability

No new data were created or analyzed in this study. Data sharing is not applicable to this article.
